# Human glutathione transferases catalyze the reaction between glutathione and nitrooleic acid

**DOI:** 10.1016/j.jbc.2025.108362

**Published:** 2025-02-28

**Authors:** Martina Steglich, Nicole Larrieux, Ari Zeida, Joaquín Dalla Rizza, Sonia R. Salvatore, Mariana Bonilla, Matías N. Möller, Alejandro Buschiazzo, Beatriz Alvarez, Francisco J. Schopfer, Lucía Turell

**Affiliations:** 1 Laboratorio de Enzimología, Instituto de Química Biológica, Facultad de Ciencias, Universidad de la República, Montevideo, Uruguay; 2 Centro de Investigaciones Biomédicas (CEINBIO), Universidad de la República, Montevideo, Uruguay; 3 Graduate Program in Chemistry, Facultad de Química, Universidad de la República, Montevideo, Uruguay; 4 Unidad de Cristalografía de Proteínas, Institut Pasteur de Montevideo, Montevideo, Uruguay; 5 Facultad de Medicina, Departamento de Bioquímica, Universidad de la República, Montevideo, Uruguay; 6 Department of Pharmacology and Chemical Biology, University of Pittsburgh School of Medicine, Pittsburgh, USA; 7 Laboratorio de Biología Redox de Tripanosomas, Institut Pasteur de Montevideo, Montevideo, Uruguay; 8 Laboratorio de Fisicoquímica Biológica, Instituto de Química Biológica, Facultad de Ciencias, Universidad de la República, Montevideo, Uruguay; 9 Pittsburgh Heart, Lung and Blood Vascular Medicine Institute, University of Pittsburgh, Pittsburgh, Pennsylvania, USA; 10 Pittsburgh Liver Research Center, University of Pittsburgh, Pittsburgh, Pennsylvania, USA

**Keywords:** glutathione transferase, nitrooleic acid, glutathione, adduct, enzyme kinetics, thiol, crystal structure

## Abstract

Nitroalkene fatty acids (NO_2_-FAs) are formed endogenously. They regulate cell signaling pathways and are being developed clinically to treat inflammatory diseases. NO_2_-FAs are electrophilic and form thioether adducts with glutathione (GSH), which are exported from cells. Glutathione transferases (GSTs), a superfamily of enzymes, contribute to the cellular detoxification of hydrophobic electrophiles by catalyzing their conjugation to GSH. Herein, we evaluated the capacity of five human GSTs (M1-1, M2-2, M4-4, A4-4, and P1-1) to catalyze the reaction between nitrooleic acid (NO_2_-OA) and GSH. The reaction was monitored by HPLC-ESI-MS/MS, and catalytic activity was detected with hGSTs M1-1 and A4-4. Using stopped-flow spectrophotometry, a 1400- and 7500-fold increase in the apparent second-order rate constant was observed for hGST M1-1 and hGST A4-4, respectively, compared to the uncatalyzed reaction (pH 7.4, 25 °C). The acceleration was in part due to a higher availability of the thiolate. The crystal structure of hGST M1-1 in complex with the adduct was solved at 2.55 Å resolution, revealing that the ligand was bound within the active site, and establishing a foundation to build a model of hGST A4-4 in complex with the adduct. A larger number of interactions between the enzyme and the fatty acid were observed for hGST A4-4 compared to hGST M1-1, probably contributing to the increased catalysis. Altogether, these results show, for the first time, that hGSTs can catalyze the reaction between GSH and NO_2_-FAs, likely affecting the signaling actions of these metabolites and expanding the repertoire of GST substrates.

Nitroalkene fatty acids (NO_2_-FAs) are electrophilic compounds that exert pleiotropic signaling actions with cytoprotective and anti-inflammatory effects in humans and rodents. *In vivo*, their formation starts with the addition of nitrogen dioxide (NO_2_^⋅^) to unsaturated fatty acids. Nitration is favored in the gastric compartment where the acidic pH leads to the protonation of nitrite (NO_2_^-^) coming from the diet or saliva, yielding nitrous acid (HNO_2_), which decomposes to NO_2_^⋅^ (1, 2, 3). Nitration also occurs in the context of inflammation where NO_2_^⋅^ production is increased (3, 4). Due to their beneficial therapeutic properties, NO_2_-FAs have been tested as a treatment for various diseases. Nitrooleic acid (NO_2_-OA), the nitro derivative of oleic acid, has been used as a model to study NO_2_-FAs metabolism, pharmacokinetics, and pharmacodynamics. It has shown promising effects as a potential drug candidate and is being developed clinically mainly to treat inflammatory and cardiovascular diseases (5, 6, 7, 8, 9, 10, 11). Two regioisomers, 9- and 10-NO_2_-OA, can be formed depending on which carbon atom undergoes nitration (12, 13).

As electrophiles, NO_2_-FAs undergo reversible Michael addition reactions with nucleophiles, such as thiols. The formation of adducts with proteins that participate in cell signaling pathways can have functional implications, ultimately modifying patterns of gene expression that lead to the anti-inflammatory and cytoprotective properties of NO_2_-FAs. Some reported proteins that become nitroalkylated are nuclear factor kappa B (NF-κB) p65 subunit (14, 15), Kelch-like ECH-associating protein 1 (Keap1) (15, 16, 17), heat shock proteins (HSPs) (18), peroxisome proliferator-activated receptor γ (PPARγ) (19), stimulator of interferon genes (STING) (4), the 26S proteasome (20), and RAD51 recombinase (21). The mentioned proteins are not specific for particular diseases but are involved in broad disease categories through their roles in key cellular processes, such as inflammation (NF-κB) (22), stress responses (Keap1, HSPs) (23, 24), metabolism (PPARγ) (25), DNA repair (RAD recombinase) (26), and antiviral defense (STING) (27).

NO_2_-FAs also react reversibly with low molecular weight thiols. Reduced glutathione (GSH) is considered to be one of the main targets, as its intracellular levels are in the mM range (28, 29). The reaction between NO_2_-OA and GSH (Fig. 1) presents monophasic kinetics, consistent with the presence of only one electrophilic carbon, the C_β_ of the nitroalkene functional group (30, 31). The reaction proceeds through a stepwise mechanism that starts with the nucleophilic attack of the thiolate on the nitroalkene to give a nitronate intermediate. This rate-limiting step is followed by the incorporation of a proton to finally yield a GS-NO_2_-OA adduct (Fig. S1) (31, 32). The second-order rate constant for the addition reaction (*k*_on_) is 64 M^-1^ s^-1^, while the first-order rate constant for the elimination reaction (*k*_off_) is 6 × 10^-3^ s^-1^ (pH 7.4, 25 °C) (31). In cells, adducts formed by the reaction of NO_2_-FAs and GSH are exported to the extracellular milieu by multidrug resistant proteins (33). After entering the circulation, the adducts are processed and eventually eliminated in the urine as cysteine and N-acetylcysteine adducts (34, 35, 36, 37). This constitutes an important pathway of NO_2_-FAs inactivation that modulates their intracellular levels (33, 38, 39). Additionally, NO_2_-FAs can undergo several other processes including metabolic oxidation, reduction, esterification, nitric oxide release, and partitioning into hydrophobic compartments (32).

**Figure 1:**
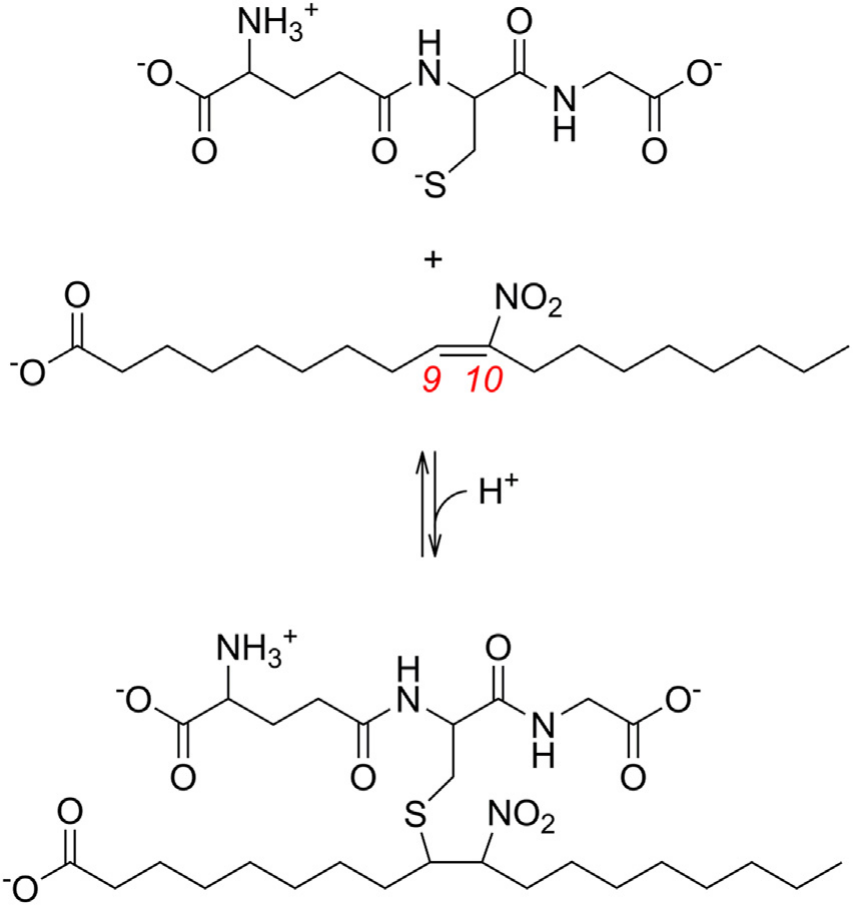
**Reversible Michael addition-elimination reaction between (*E*)-10-NO_2_-OA and GSH.** An analogous reaction can occur with (*E*)-9-NO_2_-OA. The standard numbering of the carbons of the fatty acid are shown in *red* and *italics*.

Glutathione transferases (GSTs) constitute a superfamily of enzymes (EC 2.5.1.18) involved in the metabolism of endogenous and exogenous molecules, widely distributed in nature. They are bisubstratic enzymes mostly known for conjugating GSH to electrophilic molecules, thus increasing their hydrophilicity and facilitating their elimination from the body. Other reactions catalyzed by GSTs include Michael additions, thiol disulfide exchange, and isomerization of unsaturated compounds (40, 41). According to the intracellular localization, three GST families can be distinguished: cytosolic, mitochondrial, and membrane associated. Cytosolic GSTs are divided in different classes according to their primary sequence and catalytic residues: Mu (M), Alpha (A), Pi (P), Theta (T), Sigma (S), Zeta (Z), and Omega (O) (42), which are expressed differentially in several tissues (43). Cytosolic GSTs are dimeric enzymes that bear a GSH-binding site (a conserved site known as the G-site) and a binding site for an often-hydrophobic cosubstrate (H-site), in each monomer. The latter is highly variable and accounts for the diversity of electrophilic molecules that are substrates of these enzymes (40, 41). Mitochondrial GSTs are exclusively of the Kappa (K) class. On the other hand, membrane-associated GSTs (MAPEG family) include six key enzymes in mammals and are structurally diverse trimeric transmembrane proteins (41).

In this work, we evaluated whether the reaction between NO_2_-OA and GSH was catalyzed by human GSTs (hGSTs). A previous report (38) proposed that NO_2_-FAs were able to interact with a set of human GSTs, inhibiting their activity with the canonical substrate, 1-chloro-2,4-dinitrobenzene (CDNB), although catalysis of the reaction between GSH and NO_2_-FAs was not observed then. We now tested five cytosolic GSTs *in vitro*, using NO_2_-OA and measuring the formation of the GS-NO_2_-OA adduct by high-performance liquid chromatography-electrospray ionization-tandem mass spectrometry (HPLC-ESI-MS/MS). Two variants, hGST M1-1 and hGST A4-4, were the most active in catalyzing the reaction. Kinetic analyses performed by stopped-flow spectrophotometry monitoring NO_2_-OA consumption showed that hGST A4-4 accelerates the reaction 5 to 10 times more than hGST M1-1, depending on the NO_2_-OA isomer. Moreover, we obtained the crystal structure of hGST M1-1 with the GS-10-NO_2_-OA adduct bound within the active site, and we built a model of hGST A4-4 in complex with the adduct. These findings contribute to understand the metabolism of NO_2_-OA in a cellular context and expand the range of possible substrates for GSTs.

## Results

### Screening of hGSTs for the catalysis of the reaction between GSH and NO_2_-OA

**Figure 2:**
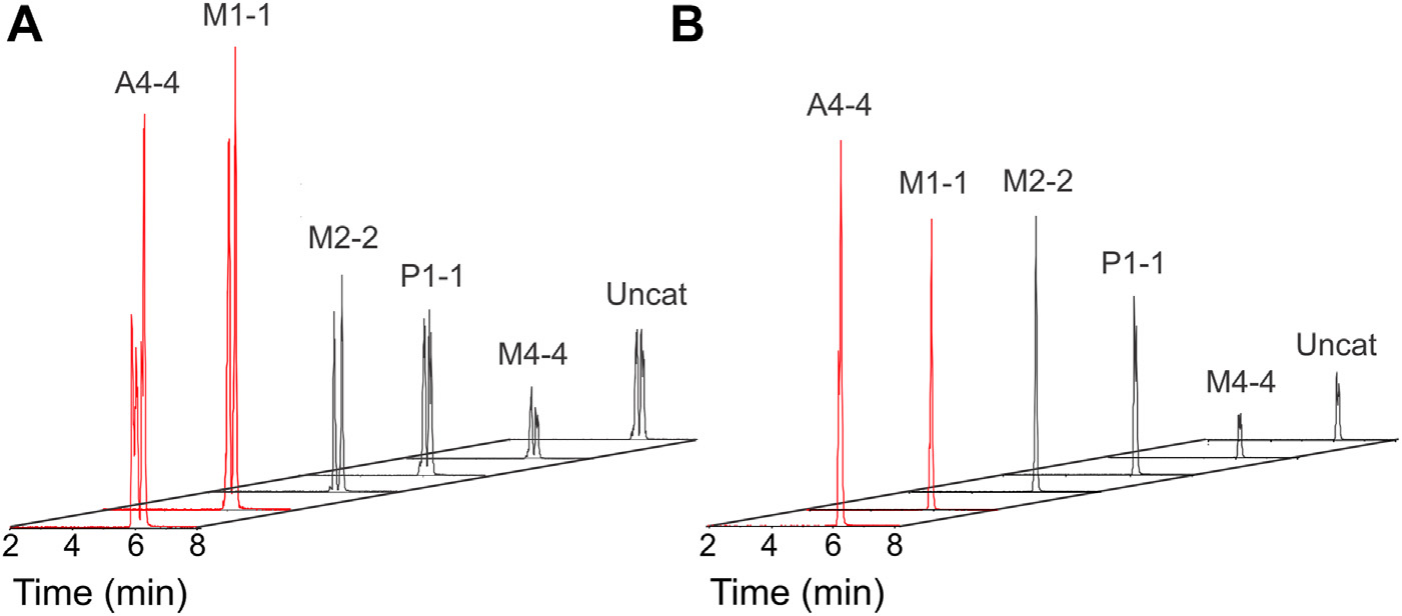
**Screening by HPLC-ESI-MS/MS of the reaction between NO_2_-OA and GSH in the presence of hGSTs.***A*, an equimolar mixture of 9- and 10-NO_2_-OA or (*B*) 10-NO_2_-OA (2 μM) and GSH (200 μM) were mixed in the absence (uncatalyzed) or presence of five commercially available hGSTs (hGST M1-1, M2-2, M4-4, A4-4, and P1-1) in phosphate buffer (20 mM, pH 7.4, 25 °C). The reactions were stopped after 5 min, chromatographically resolved, and GS-NO_2_-OA formation was monitored (635.3/506.2 MRM transition).

Five commercially available hGSTs from different classes were evaluated for their ability to catalyze the reaction between GSH and NO_2_-OA; three mu-type (hGST M1-1, M2-2, and M4-4), one alpha (hGST A4-4), and one pi (hGST P1-1). These isoforms were selected based on our previous results with a mu-type enzyme from another organism (manuscript in preparation), the reported Michael addition of 4-hydroxynonenal catalyzed by hGST A4-4 (44, 45, 46, 47), and the clinical relevance of hGST P1-1 in cancer development and treatment (48, 49). Reaction mixtures were prepared containing GSH (200 μM) and an equimolar mixture of 9- and 10-NO_2_-OA (Fig. 2*A*) or 10-NO_2_-OA (2 μM) (Fig. 2*B*), in the absence or presence of the different enzymes (0.07 μM). GS-NO_2_-OA adduct formation was evaluated by the 635.3/506.2 multiple reaction monitoring (MRM) transition after 5 min of reaction, by HPLC-ESI-MS/MS. Increased levels of adduct formation were observed for all hGSTs, except for M4-4. The enzymes hGST M1-1 and hGST A4-4 showed more product formation using both the equimolar mixture of 9- and 10-NO_2_-OA and the purified 10-NO_2_-OA. The profile obtained for the GS-NO_2_-OA adduct obtained when using the purified 10-NO_2_-OA was simpler, as a smaller number of isomers was obtained. Based on these primary findings, hGST M1-1 and hGST A4-4 were selected for further characterization, thus they were produced recombinantly in-house.

### Expression, purification, and characterization of recombinant hGST M1-1 and hGST A4-4

**Figure 3:**
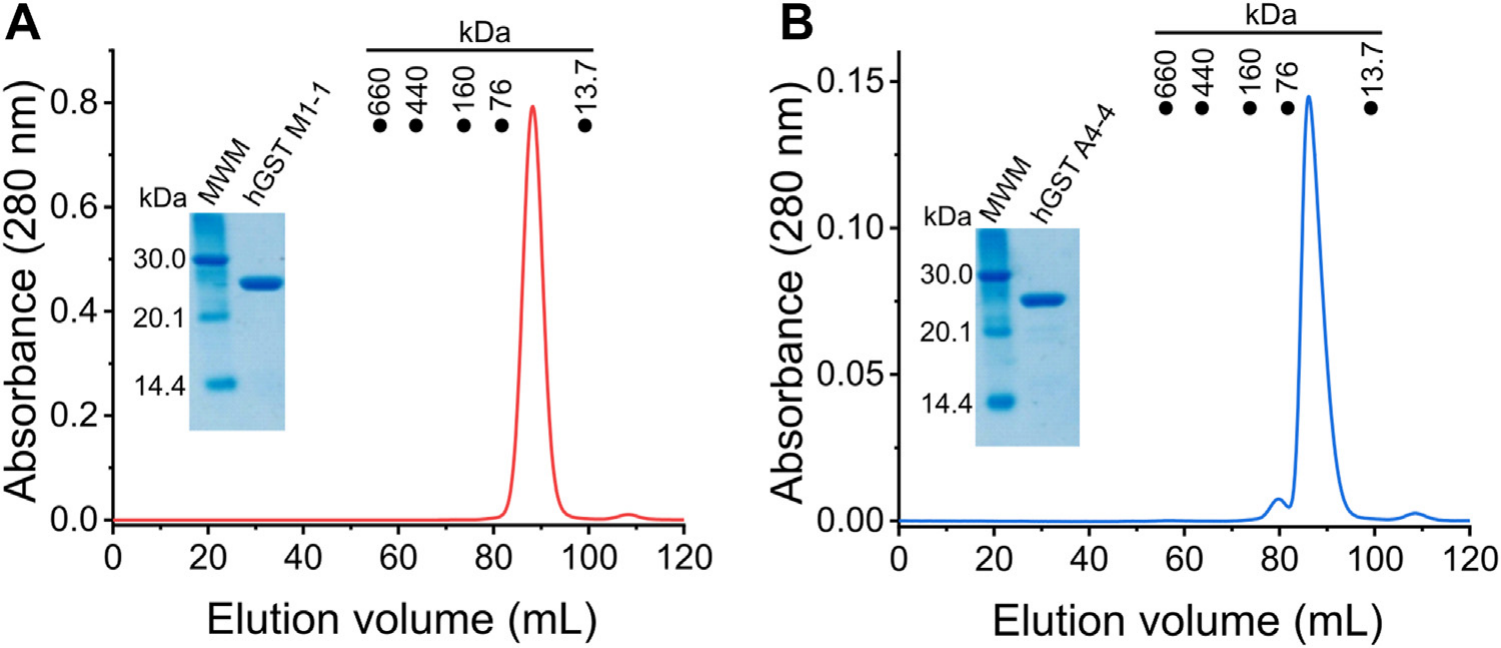
**Final purification step of recombinant hGSTs and SDS-PAGE analysis.** Size exclusion chromatography of (*A*) hGST M1-1 and (*B*) hGST A4-4 was performed using a HiLoad 16/600 Superdex 200. The closed circles indicate the elution volume of the proteins used in the calibration (thyroglobulin, 660 kDa; ferritin, 440 kDa; aldose, 160 kDa; conalbumin, 76 kDa; and ribonuclease A, 13.7 kDa). Inset, reducing SDS-PAGE after SEC (4 μg hGST).

Both enzymes were expressed and purified as described in Experimental procedures. hGST M1-1 was purified without tags, by affinity chromatography on GSH-Sepharose, with excellent yield (∼50 mg per L of culture) and purity (>99%), according to SDS-PAGE and size-exclusion chromatography (SEC) (Fig. 3*A*). The identity was confirmed by MS analysis of tryptic fragments (Uniprot ID P09488, canonical sequence). A dimeric quaternary structure was suggested by SEC (expected: ∼51 kDa). A specific activity of ∼212 μmol min^-1^ mg^-1^ was obtained using the canonical substrates, GSH and CDNB, in good agreement with reported values (38, 50). Using 5,5′-dithiobis(2-nitrobenzoate) (DTNB) (51), four thiols were quantified per monomer of hGST M1-1, as expected from its sequence.

The expression of hGST A4-4 was first attempted using the same strategy as for hGST M1-1, but neither soluble nor insoluble protein was obtained. To overcome this drawback, hGST A4-4 was expressed as a C-terminal fusion to a His-tagged thioredoxin 1 (Trx1) with a tobacco etch virus (TEV) protease cleavage site in between. This led to excellent yields (∼60 mg/L) and purity (>98%) after TEV protease cleavage (Fig. 3*B*). The identity was confirmed by MS (Uniprot ID O15217, canonical sequence), and SEC suggested hGST A4-4 was also a dimer (expected: ∼52 kDa). The specific activity was ∼9 μmol min^-1^ mg^-1^, using GSH and CDNB as substrates, consistent with reported values (38, 44). hGST A4-4 does not contain any cysteines in its primary sequence.

### HPLC-ESI-MS/MS and HPLC-UV-Vis assessment of the reaction between GSH and NO_2_-OA catalyzed by hGST M1-1 and hGST A4-4

**Figure 4:**
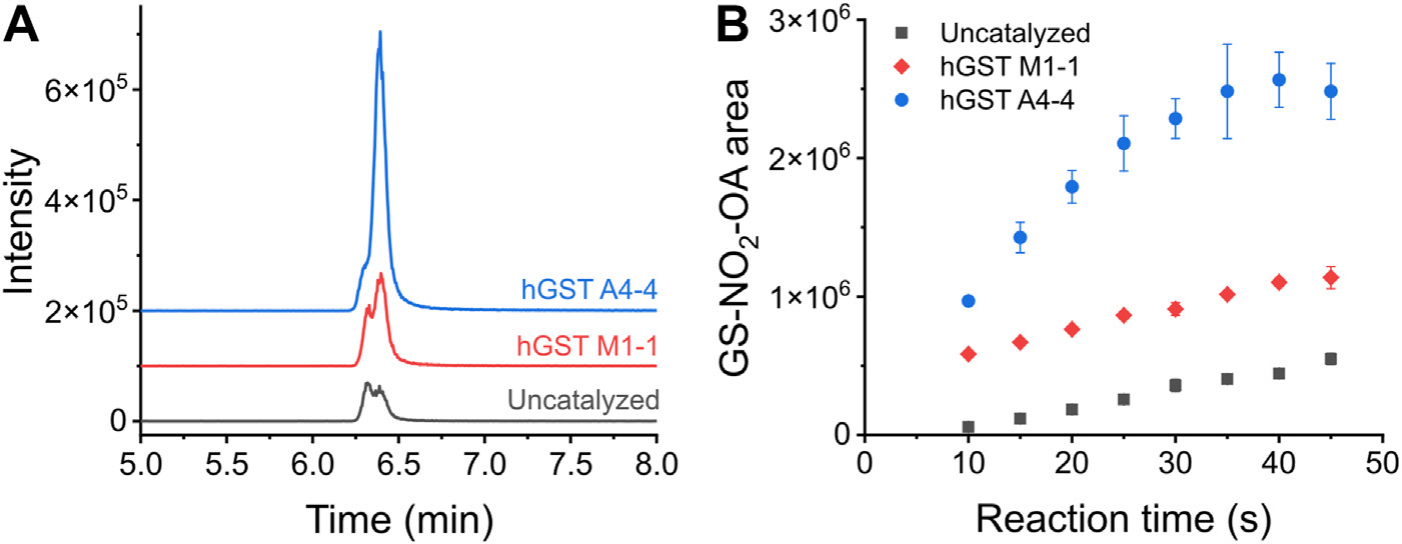
**Evaluation by HPLC-ESI-MS/MS of the reaction between NO_2_-OA and GSH in the presence of hGST M1-1 and hGST A4-4.** 10-NO_2_-OA (2 μM) and GSH (200 μM) were mixed in the absence or presence of recombinant hGST M1-1 and hGST A4-4 purified in-house (0.07 μM), in phosphate buffer (20 mM, pH 7.4, 25 °C). Aliquots were taken at increasing times for 45 s and the reactions were immediately stopped. *A*, representative GS-NO_2_-OA adduct profiles obtained at 45 s of reaction. *B*, areas of the GS-NO_2_-OA adduct formed at increasing reaction times. The symbols represent the mean ± SD, n = 3. Some error bars are smaller than the symbols.

HPLC-ESI-MS/MS results presented above (Fig. 2) correspond to a preliminary screening. A more detailed study of the reaction was performed using hGST M1-1 or hGST A4-4 (expressed in-house) and the 10-NO_2_-OA isomer to simplify the analysis. Mixtures containing 10-NO_2_-OA (2 μM) and GSH (200 μM) in the absence and presence of hGST M1-1 or hGST A4-4 (0.07 µM) were incubated for 45 s. An aliquot of the product mixture was analyzed by HPLC-ESI-MS/MS. By monitoring the formation of the adduct, two overlapping peaks were obtained (Fig. 4*A*), probably corresponding to stereoisomers of the GS-NO_2_-OA adduct. To evaluate the progression of the reactions, time course analysis was performed, and the total area of the peak corresponding to the adduct was measured. The adduct was formed faster by both enzymes with higher rates for hGST A4-4. No more product was formed after 40 s in the presence of hGST A4-4, while product was still being formed moderately after 45 s in the presence of hGST M1-1 and in the absence of enzyme (uncatalyzed reaction) (Fig. 4*B*).

**Figure 5:**
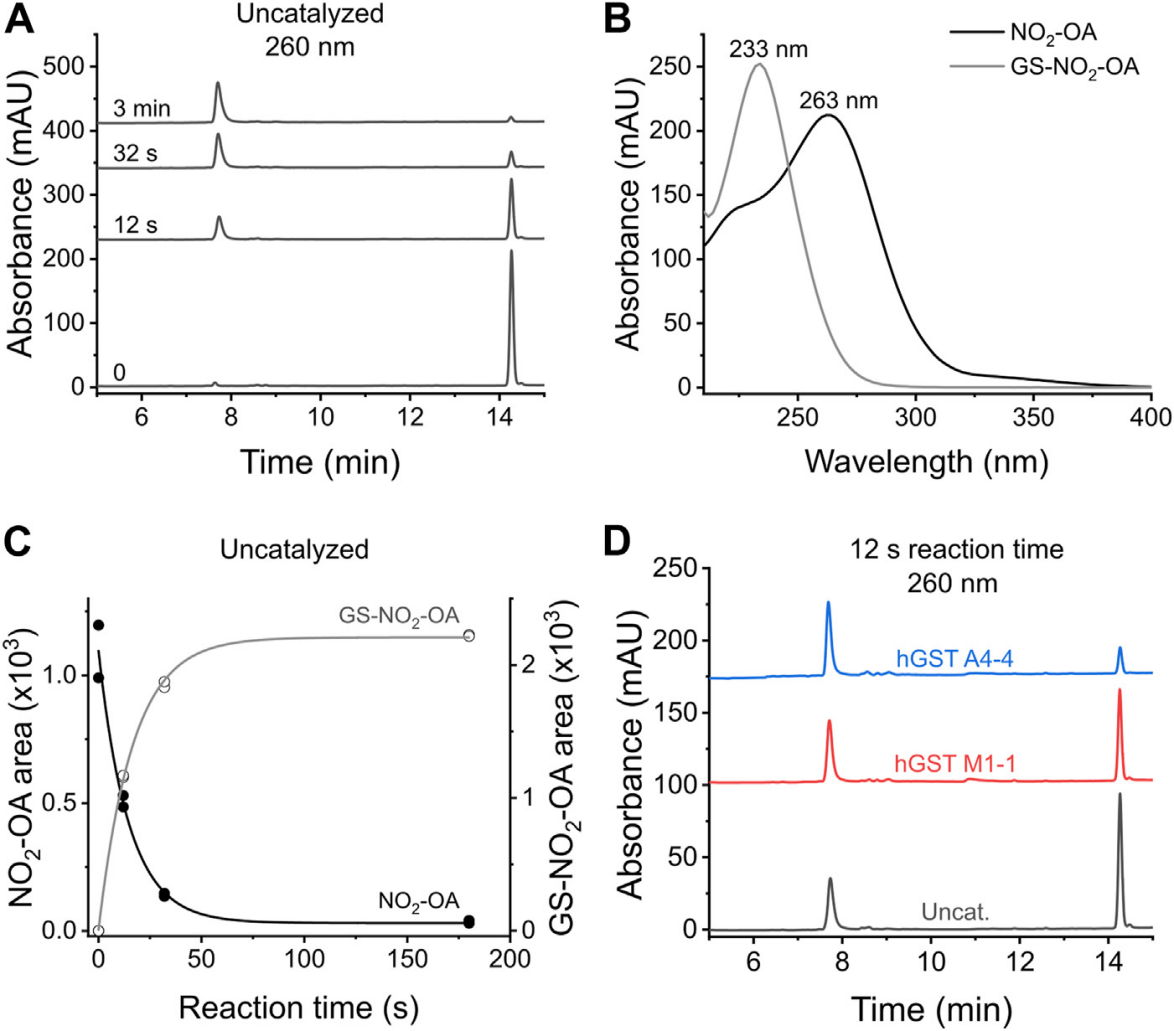
**HPLC-UV-Vis analysis of the reaction between GSH and NO_2_-OA.***A*, chromatograms followed by absorbance at 260 nm obtained for reaction mixture aliquots (10 μl) of 10-NO_2_-OA (68 μM) before (time zero) and after GSH (2 mM) addition (12 s, 32 s, and 3 min) in phosphate buffer (20 mM, pH 7.4, 25 °C). The small peak observed at time zero with a retention time of 7.6 min does not correspond to the GS-NO_2_-OA adduct (7.7 min), as it presents different retention time and spectral properties, and it is present in the baseline. *B*, UV-Vis spectra of the peaks corresponding to retention times of 7.7 min (GS-NO_2_-OA adduct, λ_max_ 233 nm) and 14.3 min (NO_2_-OA, λ_max_ 263 nm). *C*, calculated areas for both species, NO_2_-OA and GS-NO_2_-OA adduct, at increasing times. *D*, chromatograms at 260 nm obtained for aliquots (10 μl) of the uncatalyzed and catalyzed reactions with hGST M1-1 (2.5 μM) or hGST A4-4 (1 μM), after 12 s of reaction.

The formation of the GS-NO_2_-OA adduct concomitant with NO_2_-OA consumption was analyzed by chromatographic separation of the species by HPLC and assessed by UV-Vis absorbance. First, the uncatalyzed reaction was evaluated. 10-NO_2_-OA (68 μM) was mixed with GSH (2 mM) and aliquots were analyzed at increasing times. The initial NO_2_-OA present in the mixture before GSH addition was also measured (time zero). Only one peak was observed in the absence of GSH corresponding to authentic NO_2_-OA, with a retention time of 14.3 min and an absorbance maximum at 263 nm (Fig. 5, *A* and *B*). When GSH was added, a peak with a retention time of 7.7 min corresponding to the GS-NO_2_-OA adduct was identified, with a blue-shifted maximum at 233 nm. The decrease in retention time compared to the one of NO_2_-OA is explained by the decreased hydrophobicity of the adduct, as it contains a GSH moiety. The blue shift observed in the absorbance maximum is due to the disruption of the conjugated π-system caused by the addition of GSH (Fig. 5, *A* and *B*). As expected, the adduct was formed at the expense of NO_2_-OA. From the time course of adduct formation, an observed exponential rate constant (*k*_obs_) of 0.07 ± 0.01 s^-1^ could be estimated (mean ± error of the fit, n = 2) (Fig. 5*C*). Assuming a negligible reverse reaction, this *k*_obs_ translates into a second-order rate constant of 35 ± 5 M^-1^ s^-1^, consistent with previous reports (31).

To evaluate any possible profile or product differences between the catalyzed and uncatalyzed reactions, reaction products were compared within a short reaction time (12 s) (Fig. 5*D*). The same peaks were observed as for the uncatalyzed reaction. An increase in adduct formation and NO_2_-OA consumption was observed for both enzymes. As observed by HPLC-ESI-MS/MS (Fig. 4), the reaction catalyzed by hGST A4-4 was faster than the one catalyzed by hGST M1-1.

### Stopped-flow spectrophotometric analysis of the reaction between NO_2_-OA and GSH in the presence of hGSTs

**Figure 6:**
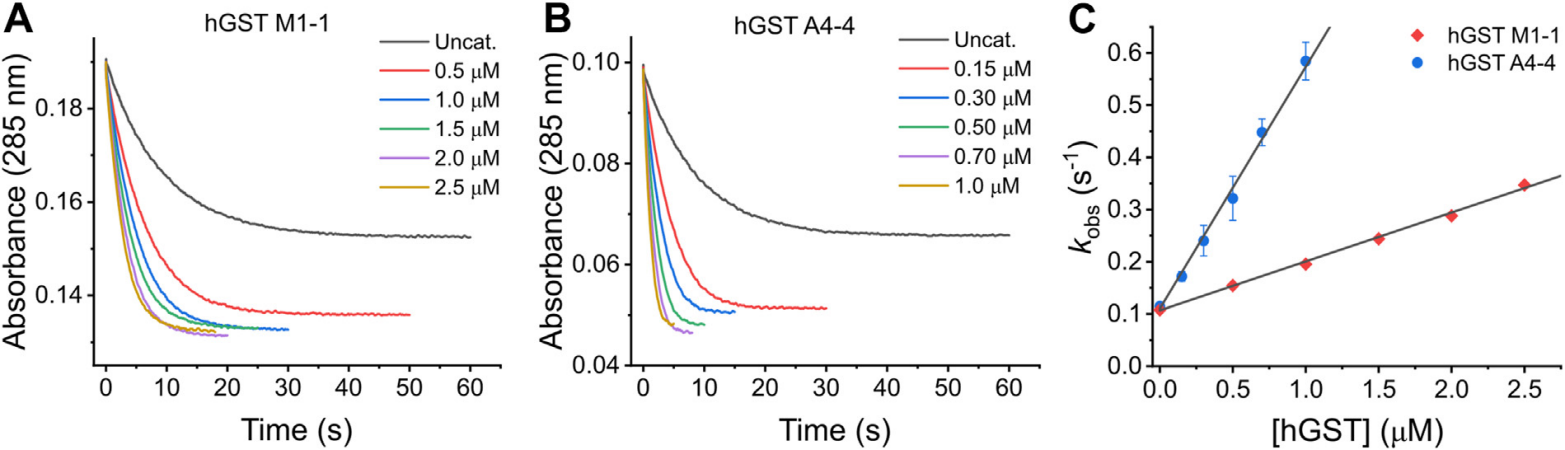
**Kinetic characterization of the reaction between NO_2_-OA and GSH catalyzed by hGST M1-1 and hGST A4-4.** An equimolar mixture of 9- and 10-NO_2_-OA (20 μM) was mixed with GSH (2 mM) in the absence or presence of (*A*) hGST M1-1 (0.5–2.5 μM) or (*B*) hGST A4-4 (0.15–1.0 μM) in phosphate buffer (100 mM pH 7.4, 0.1 mM DTPA, 25 °*C*), and the absorbance at 285 nm was registered. An average time course (n = 4) is shown for each hGST concentration. For comparison, time courses were set to start at the same point in the absorbance scale, considering that the enzyme also absorbs. *C*, *k*_obs_ values were determined from the fit of the nonaveraged data in (*A*) or (*B*) to exponential functions and plotted against hGST concentration. The symbols represent the mean ± SD, n = 4. Some error bars are smaller than the symbols.

Stopped-flow experiments were performed to study the kinetics of the reaction with both hGSTs quantitatively. An equimolar mixture of 9- and 10-NO_2_-OA (20 μM) was mixed with GSH in pseudo-first order excess (2 mM), in the absence and presence of increasing concentrations of hGST M1-1 or hGST A4-4. Monophasic kinetics were observed for both enzymes, and for the uncatalyzed reaction (Fig. 6, *A* and *B*), in agreement with NO_2_-OA having only one electrophilic site for the formation of adducts with GSH. An increase in the total change in absorbance was observed in the presence of hGSTs suggesting that more adduct was being formed at the end of the reaction, compared to the uncatalyzed one. This was unexpected as enzymes do not change the position of the equilibrium. In addition, the increase was not proportional to enzyme concentration. The origin of this increase in the total change in absorbance is yet unclear. Controls in the absence of GSH showed no consumption of NO_2_-OA in the assay conditions, ruling out any reaction with surface or interior nucleophilic residues (histidines or reduced cysteines) in the enzymes (Fig. S2).

Single exponential or exponential plus straight-line functions were reliably fitted to the time course curves, from which the *k*_obs_ were obtained for each condition. A linear dependence of *k*_obs_ with enzyme concentration was observed for both hGSTs, consistent with the existence of catalysis (Fig. 6*C*). In this system, the *k*_obs_ can be defined as the sum of the uncatalyzed and the catalyzed apparent rate constants. If the reverse reactions are also considered, then *k*_obs_ can be described according to Equation 1:

(Eq. 1) $$k_{obs}=k_{off\;uncat}+k_{on\;uncat}\left[GSH\right]+\left(k_{off\;cat}^{app}+k_{on\;cat}^{app}\right)\left[GST\right]$$

According to this equation, the *y*-axis intercept ($k_{off\;uncat}+k_{on\;uncat}\left[GSH\right])$ corresponds to the *k*_obs_ of the uncatalyzed reaction in the presence of 2 mM GSH. The calculated value, 0.105 ± 0.002 s^-1^ (n = 3) (Fig. 6*C*), is consistent with the value obtained from HPLC-UV-Vis experiments and with previous determinations (31). The slope $\left(k_{off\;cat}^{app}+k_{on\;cat}^{app}\right)$ represented in Equation 1 presents a complex dependency on GSH and NO_2_-OA concentrations. The ratio between the slope (M^-1^ s^-1^) and the *y*-axis intercept (s^-1^) corresponds to an apparent acceleration per molar of enzyme (M^-1^) at a fixed 2 mM GSH concentration (Equation 2).

(Eq. 2) $${Acceleration\;}_{app}\left(M^{-1}\right)=\frac{k_{off\;cat}^{app}+k_{on\;cat}^{app}\;}{k_{off\;uncat}+k_{on\;uncat}\left[GSH\right]}$$

The calculated apparent acceleration was (8.3 ± 0.9) × 10^5^ M^-1^ for hGST M1-1 and (4.7 ± 0.7) × 10^6^ M^-1^ for hGST A4-4, meaning that hGST A4-4 accelerates the reaction ∼6 times more than hGST M1-1 (Fig. 6*C*). When 10-NO_2_-OA was used (Fig. S3) instead of the equimolar mixture of 9- and 10-NO_2_-OA, a slight decrease in the acceleration was observed for hGST M1-1 ((2.9 ± 0.6) × 10^5^ M^-1^), probably due to preference of hGST M1-1 for the 9-NO_2_-OA regioisomer. For hGST A4-4, the acceleration obtained with 10-NO_2_-OA ((3 ± 1) × 10^6^ M^-1^) was similar to that obtained with the equimolar mixture of 9- and 10-NO_2_-OA.

### p*K*_*a*_ of GSH bound to hGSTs

**Figure 7:**
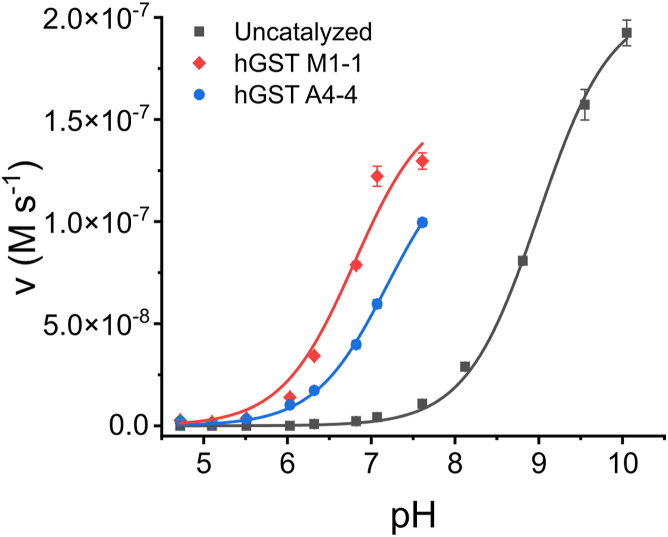
**pH dependence of the reaction rates between CDNB and GSH in the absence or presence of hGST M1-1 and hGST A4-4.** The initial rate of the reaction between GSH (2 mM) and CDNB (75 μM) was measured in the absence (uncatalyzed) and presence of hGST M1-1 (1.4 nM) or hGST A4-4 (14 nM), in three-component buffer (0.1 M MES, 0.052 M Tris, 0.052 M ethanolamine) of varying pH. The rates of the uncatalyzed reaction were subtracted from those in the presence of the enzyme. The *solid* lines represent the best fit of Equation 3 to the data. The symbols represent the mean ± SD, n = 3. Some error bars are smaller than the symbols.

The reactive form of GSH is the thiolate (GS^-^). The proportion of GS^-^ depends on the p*K*_*a*_ and the pH of the solution. It has been previously reported that one of the mechanisms by which GSTs catalyze the nucleophilic attack of GS^-^ on electrophiles is by lowering the p*K*_*a*_ of the GSH bound in the active site to increase the proportion of GS^-^ (52, 53). To determine the p*K*_*a*_ of GSH bound to hGST M1-1 and hGST A4-4, the canonical substrate CDNB was used. Unlike NO_2_-OA, CDNB is amenable to typical steady-state measurements. In addition, CDNB has no ionizable groups, is stable at different pHs, has relatively high solubility, its reaction product with GSH has a known absorption coefficient, and is irreversible. The reaction between CDNB and GSH is a nucleophilic aromatic substitution in which GS^-^ attacks the electrophilic carbon of the carbon-chlorine bond in CDNB (Fig. S4). The initial, steady-state, reaction rates were measured at different pHs with CDNB (75 μM) and GSH (2 mM) in the absence or presence of hGST M1-1 (1.4 nM) or hGST A4-4 (14 nM), using a three-component buffer system with constant ionic strength (54). Under these conditions, the initial rate of the catalyzed reaction is proportional to the GSH-saturated enzyme and free CDNB, and its pH-dependence reflects the ionization that occurs in enzyme-bound GSH. Initial rates (*v*) for the three conditions assayed were plotted against pH and fitted to Equation 3 (Fig. 7), yielding the pH-independent rate (when all the GSH is ionized, v_pH ind_) and the p*K*_*a*_ of free and enzyme-bound GSH.

(Eq. 3) $$v=\frac{v_{pH\;ind}\times 10^{-pKa}}{10^{-pKa}+10^{-pH}}$$

The v_pH ind_ was (2.06 ± 0.04) × 10^-7^ M s^-1^ for the uncatalyzed reaction, while in the presence of hGST M1-1 and hGST A4-4, v_pH ind_ were (1.59 ± 0.05) × 10^-7^ M s^-1^ and (1.37 ± 0.09) × 10^-7^ M s^-1^, respectively. In the absence of enzyme, a p*K*_*a*_ of 9.00 ± 0.03 was obtained for GSH, consistent with data from the literature (52, 55), while p*K*_*a*_s of 6.80 ± 0.04 and 7.19 ± 0.06 were determined for GSH bound to hGST M1-1 and hGST A4-4, respectively. The ratio of GS^-^ to total GSH at pH 7.4 was calculated (Equation 4) as 0.024 for free GSH, 0.80 for GSH bound to hGST M1-1, and 0.61 for GSH bound to hGST A4-4 (Table 1). These ratios also describe the situation when relatively low concentrations of NO_2_-OA react with free or enzyme-bound GSH.

(Eq. 4) $$\frac{\left[{GS}^{-}\right]}{{\left[GSH\right]}_{Total}}=\frac{10^{-pKa}}{10^{-pKa}+10^{-pH}}$$

**Table 1:** p*K*_*a*_ of GSH and rate constants for the reaction between GSH and NO_2_-OA

	Uncatalyzed	hGST M1-1	hGST A4-4
Free or GST-bound GSH^a^			
p*K*_*a*_	9.00 ± 0.03	6.80 ± 0.04	7.19 ± 0.06
[GS^-^]/[GSH]_Total_	0.024	0.80	0.61
Rate constants			
*k*_pH 7.4_ (M^-1^ s^-1^)	64 ± 1^b^	(8.8 ± 0.9) × 10^4^^c^	(4.8 ± 0.2) × 10^5^^c^
*k*_pH ind_ (M^-1^ s^-1^)^d^	2.6 × 10^3^	1.1 × 10^5^	7.8 × 10^5^

a Determined using Figure 7, Equation 3 and Equation 4 (pH 7.4, 25 °C).

b Reported in Ref (31) (pH 7.4, 25 °C).

c Apparent second-order rate constant at pH 7.4, for the reaction between NO_2_-OA and free or enzyme-bound GSH, represented by *k*_cat_/*K*^NO^_2_^-OA^. Determined using Figure 6 and Equation 6 (pH 7.4, 25 °C).

d pH-independent apparent second-order rate constant, determined using Equation 7 (25 °C).

### Kinetic analysis of the catalyzed reaction

The kinetic analysis of this system is complex due to several factors. First, there is an appreciable uncatalyzed reaction between GSH and NO_2_-OA. Second, both uncatalyzed and catalyzed reactions are reversible. Third, it is not possible to work with saturating concentrations of NO_2_-OA due to micelles formation that confounds the solution-phase reaction dynamics. Fourth, the kinetics are relatively fast, complicating the measurement of initial, steady-state, rates. Thus, conventional Michaelis–Menten analysis could not be applied to calculate the *k*_cat_ and *K*_m_ of the GST enzymes for NO_2_-OA and GSH.

Further kinetic information can be obtained from the data (Fig. 6) by making some assumptions. It is well-known that GST reactions imply the formation of ternary complexes (41). In this work, we confirmed the mechanism of hGST M1-1 and hGST A4-4 using CDNB and GSH as substrates (Figs. S5 and S6, Table S1). Our results are compatible with a random sequential equilibrium mechanism (Figs. S7 and S8 Equation S1) (56, 57) consistent with previous reports (52, 58). In our case, the binding of one substrate does not affect the binding of the other substrate (Fig. S8 Equations S2 and S3). This constitutes a particular case of random sequential equilibrium mechanism where the dissociation constants of each substrate from the corresponding binary and ternary complexes are similar (Fig. S8 Equation S4). If this behavior is extrapolated to NO_2_-OA instead of CDNB (Fig. S8 Equation S5), it can be assumed that, in our experimental conditions, the mM concentration of GSH is higher than the corresponding dissociation constant from the enzyme, while the μM concentration of NO_2_-OA is lower (Fig. S8 Equations S6 and S7); accordingly, time courses of NO_2_-OA decay were exponential as expected. Last, it can be assumed that the reverse reaction is negligible. In fact, the *k*_obs_ values obtained in the absence of enzyme (Fig. 6*C*), at a 2 mM GSH concentration, are consistent with a negligible reverse reaction (31). Furthermore, in the HPLC experiments, almost no NO_2_-OA remnant was observed after 3 min of reaction (Fig. 5, *A* and *C*).

Considering all the points mentioned above, and also that the rate of the reaction will be the sum of the catalyzed and the uncatalyzed reactions (Fig. S8 Equations S8 and S9), the expected complex equation for this system can be simplified to Equations 5 and 6

(Eq. 5) $$\left[NO_{2}˗OA\right]={\left[NO_{2}˗OA\right]}_{0}\;e^{-k_{obs}t}$$

(Eq. 6) $$k_{obs}=k_{on\;uncat}\left[GSH\right]+\frac{k_{cat}}{K^{NO_{2}-OA}}\;\left[GST\right]$$

Thus, the slope of the plot of *k*_obs_ *versus* GST concentration (Fig. 6*C*) corresponds to the apparent second-order rate constant of the reaction between GSH bound to GST (GST·GSH) and free NO_2_-OA which is represented by *k*_cat_/*K*^N^^O^^2^^-^^OA^, where *k*_cat_ is the catalytic constant and *K*^NO^^2^^-OA^ is the dissociation constant of NO_2_-OA from the ternary complex. The apparent second-order rate constants obtained were (8.8 ± 0.9) × 10^4^ M^-1^ s^-1^ for hGST M1-1 and (4.8 ± 0.2) × 10^5^ M^-1^ s^-1^ for hGST A4-4 (pH 7.4, 25 °C) (Table 1).

For the reaction between GSH and NO_2_-OA, the apparent second-order rate constants of the catalyzed reactions can be compared with the second-order rate constant of the uncatalyzed reaction under the same conditions (64 M^-1^ s^-1^ (31)). Then, a 1400-fold increase in the rate constant can be estimated for hGST M1-1 and a 7500-fold increase in the case of hGST A4-4 (pH 7.4, 25 °C).

Considering the GS^-^ to total GSH ratio (Table 1), and the rate constants determined above, it is possible to calculate the pH-independent rate constants (*k*_pH ind_) for the reaction between NO_2_-OA and GS^-^ (Equation 7). The *k*_pH ind_ values were 1.1 × 10^5^ M^-1^ s^-1^ for hGST M1-1, 7.8 × 10^5^ M^-1^ s^-1^ for hGST A4-4, and 2.6 × 10^3^ M^-1^ s^-1^ for the uncatalyzed reaction (Table 1).

(Eq. 7) $$k_{pH\;p7.4}=k_{pH\;ind}\frac{{\left[{GS}^{-}\right]}_{pH\;7.4}}{{\left[GSH\right]}_{Total}}$$

The apparent second-order rate constants of the catalyzed reactions between enzyme-bound GSH and NO_2_-OA (*k*_cat_/*K*^NO^_2_^-OA^) (Table 1) can also be compared with the corresponding rate constants with CDNB (*k*_*cat*_/*K*^CDNB^) (Table S1). hGST M1-1 had a 4-fold higher specificity for CDNB, while, remarkably, hGST A4-4 had an 80-fold higher specificity for NO_2_-OA.

### Crystal structure of hGST M1-1 in complex with the GS-10-NO_2_-OA adduct

**Figure 8:**
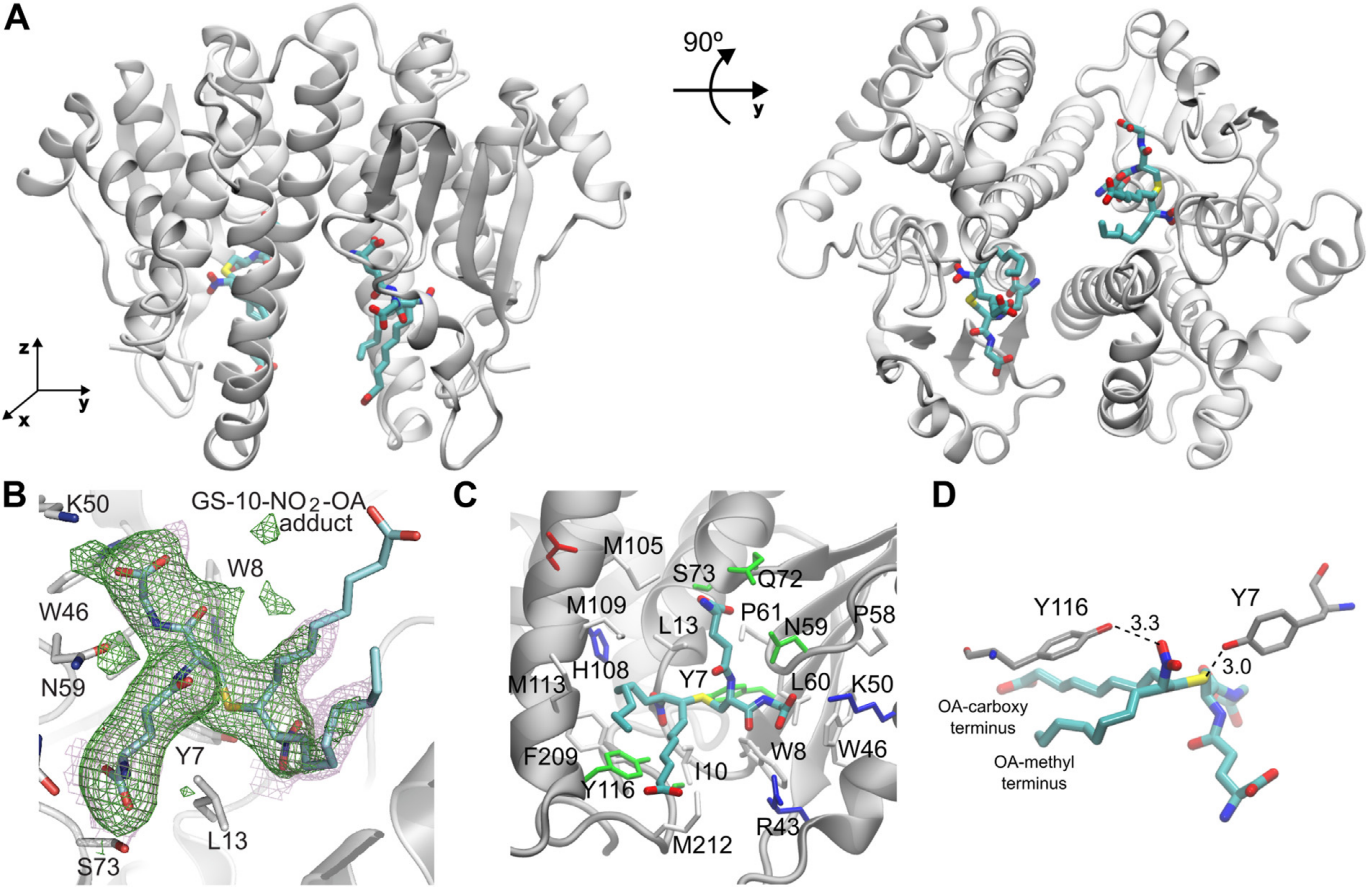
**Crystal structure of hGST M1-1 in complex with the GS-10-NO_2_-OA adduct.***A*, the hGST M1-1 dimer with the GS-10-NO_2_-OA adduct in the active site obtained by X-ray crystallography (PDB 8VOU) is shown from two different angles (90° rotation along the *y*-axis). The protein backbone is represented as a cartoon in *gray* and the ligand is represented as sticks (carbon *cyan*, oxygen *red*, nitrogen *blue*, and sulfur *yellow*). *B*, close-up on one of the GS-10-NO_2_-OA ligands (stick representation), bound to hGST M1-1 chain A within the crystal asymmetric unit. The fully refined sigmaA-weighted 2mF_obs_-DF_calc_ Fourier electron density map is shown as a *semi-transparent magenta* mesh, contoured at 1σ level (only shown around the ligand for clarity). The *green* mesh corresponds to an omit difference Fourier map calculated according to the Polder method, after removing GS-10-NO_2_-OA ligands contoured at 3σ level. A few residues close to the ligand are shown as sticks and labeled. *C*, GS-10-NO_2_-OA and amino acid sidechains within 4.5 Å. Amino acid residues are represented as sticks and colored by type (non-polar in *white*, basic in *blue*, acidic in *red*, and polar in *green*). *D*, close-up view of Y7, Y116, and the GS-10-NO_2_-OA adduct. Oleic acid carboxy and methyl termini are highlighted.

Crystal structures of hGST M1-1, including ligand-free enzyme (59, 60) as well as in complex with GSH or with products of the reaction between GSH and aromatic compounds (59, 61), have been previously reported. Attempts to obtain crystals of hGST M1-1 with 10-NO_2_-OA bound were unsuccessful, probably because NO_2_-OA concentrations had to be kept low to avoid the formation of micelles. To overcome this pitfall, we prepared the GS-10-NO_2_-OA adduct by mixing a limiting concentration of 10-NO_2_-OA with excess GSH. hGST M1-1 was crystallized in the presence of the GS-10-NO_2_-OA adduct and the structure was solved at 2.55 Å resolution (Table 2). The enzyme crystallized in the same space group as previously reported (61), with very similar unit cell parameters. With two dimers in the asymmetric unit, the refined structure displayed the typical features of hGST M1-1, including a conserved N-terminal thioredoxin-like domain (G-site) with typical βαβαββα topology and a C-terminal all-α domain (H-site) (Fig. 8*A*). The distinctive mu-loop from mu-type GSTs was observed connecting the β2 strand and the α2 helix (40, 60, 61). Structural superposition of our crystallographic model with human hGST M1-1 structures (60, 61) showed no significant differences (Fig. S9, backbone root mean squared deviation of 0.45 Å). This indicates that the presence of GS-10-NO_2_-OA in the active site does not induce significant conformational changes in the protein.

**Table 2:** Data collection and refinement statistics

Wavelength (Å)	0.77490
Resolution range (Å)	78.18–2.55 (2.59–2.55)^a^
Space group	P 2_1_ 2_1_ 2_1_
Unit cell	
a, b, c (Å)	57.38, 84.03, 213.32
Total reflections	422,093
Unique reflections	34,465 (1699)^a^
Multiplicity	12.2 (12.4)^a^
Completeness (%)	99.8 (100)^a^
Mean I/σ (I)	5.8 (0.6)^a^
Wilson B-factor	41.47
R _merge_	0.417
R _meas_	0.436
CC _1/2_	0.991 (0.314)^a^
R _work_	0.1947
R _free_	0.2265
Total number of nonhydrogen atoms:	
Macromolecule atoms	7216
Ligand atoms	192
Water atoms	89
RMS bond lengths (Å)	0.007
RMS bond angles (°)	0.82
Ramachandran analysis	
Favored (%)	97
Allowed (%)	3
Outliers (%)	0
Ramachandran plot outlier residues (%)	0
Clashscore	3
Average B-factor all atoms (Å^2^)	64
PDB ID	8VOU

a Statistics for the highest-resolution shell are shown in parentheses.

Indeed, the GS-10-NO_2_-OA adduct was bound within the active sites (Fig. 8, *A*, *B* and *C*) of the four chains within the asymmetric unit, all exhibiting the same binding pose. The adduct presented two chiral centers, identified as carbon 20 and carbon 31 (Fig. S10) in the crystal structure, which bind to the sulfur and the nitrogen of the nitro group, respectively. The electron density map suggested a (*R*,*S*) configuration for carbon 20 and carbon 31 (Fig. 8*B*), respectively, in the four chains of the two homodimers. No (*R*,*R*) or (*S*,*S*) configurations were observed, consistent with a previous computational analysis of the uncatalyzed reaction between a nitroalkene and methane thiolate. That study showed that the protonation of the C_α_ (carbon bound to the nitro group) is anti-periplanar to C_β_-S, meaning that only (*R*,*S*) or (*S*,*R*) stereoisomers could be formed (31).

The glutathionyl portion of the adduct was strongly bound to the protein by many interactions, including several H-bonds and salt bridges, engaging residues Y7, W8, L13, W46, K50, N59, L60, Q72, S73 (Fig. 8*C*), and D106' (from the other monomer, not shown in the figure). S73 established two H-bonds with the glutamyl carboxylate of the glutathionyl fragment, one through its sidechain OH, and the other *via* its mainchain N. It is noteworthy that S73 sits at the N-terminal tip of helix α3, pointing its positive dipole towards the ligand’s negative carboxylate. The OH group of Y7, a key residue for catalysis (59, 61), was at H-bonding distance (3.0 Å) from the sulfur of GS-10-NO_2_-OA (Fig. 8*D*).

The fatty acid portion comprising the carbon with the bound nitro group (carbon 31, Fig. S10) and the methyl terminus sat in close proximity to the hydrophobic H-site containing nonpolar residues M109, M113, and F209. On the other hand, the fatty acid portion involving the carboxy terminus sat in a hydrophobic pocket and did not appear to establish further interactions (Fig. 8*C*). A higher mobility was observed for the carboxylate compared to the rest of the structure, as evidenced by a weaker electron density (Fig. 8*B*), as well as higher atomic displacement factors (or B factors). From the opposite side to Y7, another tyrosine (Y116, on the C-terminal tip of α4) established a 3.3 Å H-bond to one of the oxygens of the fatty acid nitro group (Fig. 8*D*).

hGST M1-1 presents four cysteine residues per monomer, C78, C87, C115, and C174. In agreement with the DTNB measurements, all four cysteines were in the reduced form in the crystal, and none of them were exposed to the solvent. C115 and C174 were buried in the inside of the monomer while C78 and C87 were located on the dimer interface, 5.6 Å apart, a distance not compatible with disulfide bond formation.

### Computational model of hGST A4-4 in complex with the GS-10-NO_2_-OA adduct

**Figure 9:**
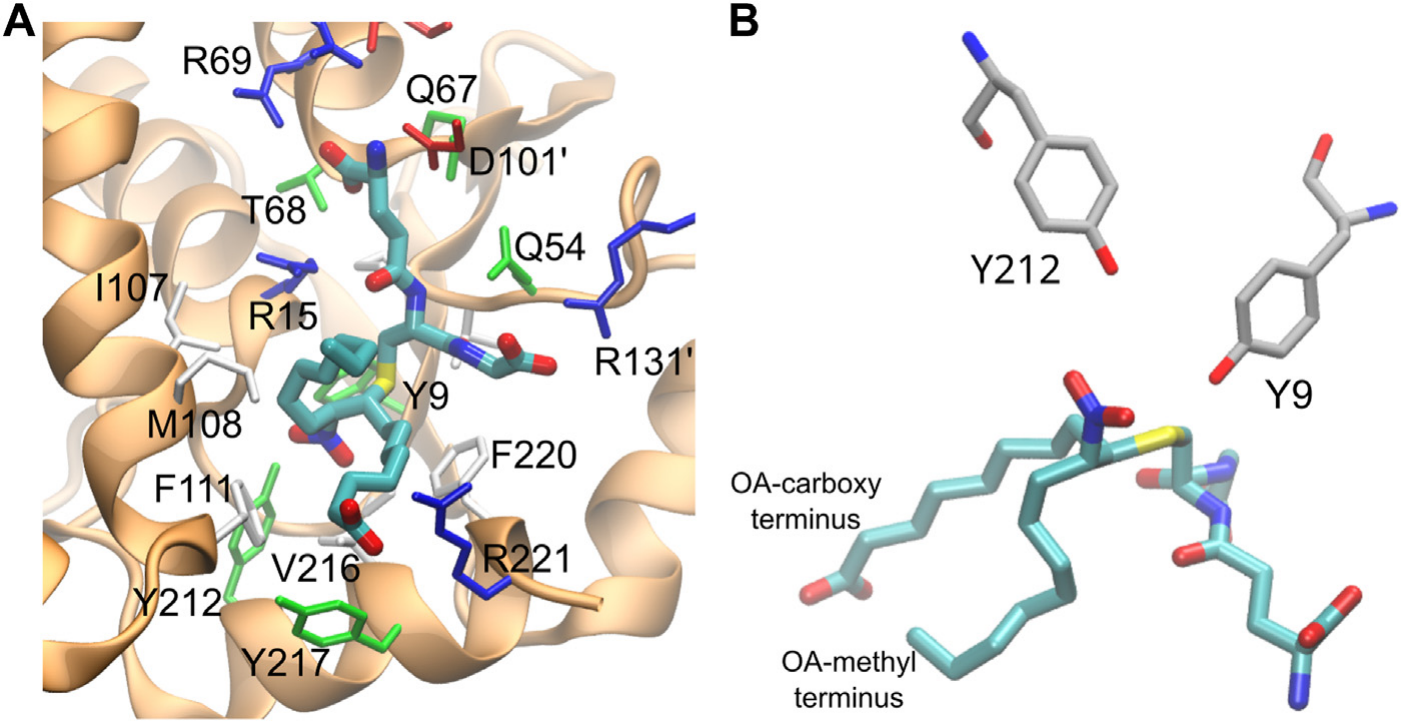
**Computational model of hGST A4-4 in complex with the GS-10-NO_2_-OA adduct.***A*, the backbone of hGST A4-4 is represented as a cartoon in *orange*. GS-10-NO_2_-OA is represented as sticks (carbon *cyan*, oxygen *red*, nitrogen *blue*, and sulfur *yellow*), and sidechains of amino acids within 4.5 Å are represented as sticks and colored by type (nonpolar residues in *white*, basic in *blue*, acidic in *red*, and polar in *green*). *B*, close-up view of Y9, Y212, and the GS-10-NO_2_-OA adduct. Oleic acid carboxy and methyl termini are highlighted.

A pairwise alignment of hGST M1-1 and hGST A4-4 protein sequences showed that both proteins share 21.3% identity and 42.6% similarity (Fig. S11). According to our kinetic results, hGST A4-4 is better than hGST M1-1 at catalyzing the reaction between GSH and NO_2_-OA. To better understand this difference, a model of the complex between hGST A4-4 and GS-10-NO_2_-OA was built (Fig. 9). Homology modeling and minimization were performed, starting from a crystal structure of hGST A4-4 (PDB 3IK7) (47) and the coordinates of hGST M1-1 complexed with GS-10-NO_2_-OA obtained in this work. The binding mode of the GSH moiety of the modeled hGST A4-4 (Fig. 9*A*) was very similar to that observed for hGST M1-1 (Fig. 8*C*). Noteworthy, the sulfur-tyrosine H-bond established by Y7 in hGST M1-1 is conserved in hGST A4-4, where Y9 most likely plays the same role (Fig. 9*B*). Intriguingly, while hGST M1-1 Y116 interacts with the nitro group of the fatty acyl moiety, hGST A4-4 has a phenylalanine (F111) in that position (Fig. S11). However, Y212 towards the C-terminus of hGST A4-4 could play an analogous role as the model predicts its close proximity to the nitro group (Fig. 9*B*).

The most conserved continuous sequence segment comparing hGST M1-1 and A4-4 (Fig. S11) is far from the reaction center, yet engaged in packing α3 in position, further supporting a role for the α3 helical dipole to properly anchor the glutathionyl moiety in the active site. hGST M1-1 S73 is substituted by a threonine (T68) in hGST A4-4, interacting similarly *via* the sidechain and mainchain with the glutamyl carboxylate. Quantitative comparison between atom distances in hGST M1-1 and hGST A4-4 was avoided because hGST A4-4 data came from homology modeling and minimization processes.

**Figure 10:**
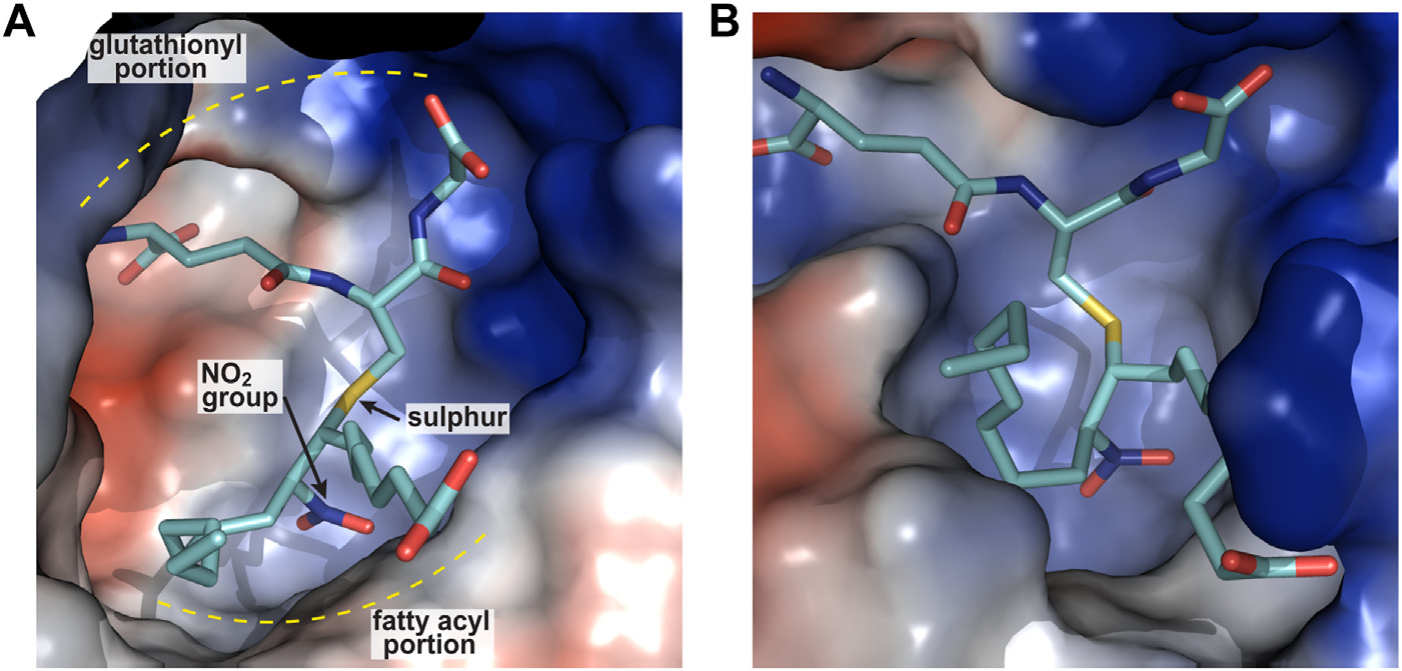
**Close-up of the reaction center of both enzymes containing the GS-10-NO_2_-OA adduct**. *A* hGST M1-1 and (*B*) hGST A4-4. The perspective is chosen so that the carboxylate-containing portion of the fatty acyl fragment is closer to the reader at the lower right part of the panels. The ligands are depicted as sticks (carbon *cyan*, oxygen *red*, nitrogen *blue*, and sulfur *yellow*). The protein is shown in molecular surface representation colored by mapping the electrostatic potential (*red* to *blue* ramp, from negative to positive).

Variations in the conformation of the fatty acid chain were observed for hGST A4-4 compared with hGST M1-1. For hGST A4-4, the portion of the carbon chain containing the carboxylate was close to the phenyl ring of Y217 and to the hydrophobic residues F111, V216, and F220. Furthermore, the location of the negatively charged carboxylate group was compatible with the establishment of a salt bridge with a positive residue, R221 (Fig. 9*A*). Altogether, these differences in the amino acids that surround the fatty acid moiety of the adduct determine that the fatty acid chain presents a loose configuration in the active site of hGST M1-1 (open pocket), while it presents a tighter fit in the active site of hGST A4-4 (closed pocket) (Fig. 10).

## Discussion

Our work shows for the first time that at least two cytosolic hGSTs from different classes, hGST M1-1 and hGST A4-4, catalyze the reaction between NO_2_-OA and GSH. The reaction progress was monitored by either product formation or NO_2_-OA consumption, using multiple methodologies including HPLC-ESI-MS/MS, HPLC-UV-Vis, and stopped-flow kinetics.

A previous report by Bates et al. (38) stated that hGSTA1-1, hGSTA4-4, hGSTM1-1, and hGSTP1-1 were unable to enhance the rate of GSH addition to NO_2_-OA. They showed only one figure on this subject, a plot of absorbance at 245 nm *versus* time in the absence and presence of hGSTs. In the light of our own results, we find that the absorbance changes reported therein were small, the time courses for the uncatalyzed reaction were too slow (much slower than can be predicted based on the rate constant), the amount of enzyme used was too low to detect changes (except maybe in the case of hGST A4-4), and the concentration of NO_2_-OA used was probably above the critical micelle concentration. Thus, we have discrepancies with the data that led to the conclusion that hGSTs did not catalyze the reaction with NO_2_-OA. Bates et al. (38) also reported that the hGST-catalyzed reaction between CDNB and GSH was inhibited by NO_2_-OA. This inhibition is consistent with the catalysis of the reaction between GSH and NO_2_-OA that we detected. Indeed, an alternative substrate behaves as a competitive inhibitor (56). However, our estimations based on the *k*_cat_/*K*^NO^_2_^-OA^ values (Fig. 6, Table 1 and Table S1) are unable to quantitatively explain the very low *K*_i_ values that Bates et al. obtained nor their finding that hGST M1-1 inhibited more than hGST A4-4. Last, the reported inhibition of the CDNB reaction with the GS-NO_2_-OA adduct (38) agrees with the fact that we were able to solve the structure of hGST M1-1 in complex with the adduct.

Our kinetic analysis (Fig. 6 and Table 1) showed that the apparent second-order rate constants for the reaction between GSH-saturated GSTs and NO_2_-OA increased 1400 times with hGST M1-1 and 7500 times with hGST A4-4, compared to the uncatalyzed reaction. In turn, the p*K*_*a*_ of GSH decreased from 9.00 ± 0.03 to 6.80 ± 0.04 and 7.19 ± 0.06 upon binding to hGST M1-1 and hGST A4-4, respectively. Does the lower p*K*_*a*_ of GSH in the presence of hGSTs account for this increase in the rate constants? The availability of GS^-^ (ratio of GS^-^ to total, Equation 4) increased only 33-fold in the presence of hGST M1-1 and 25-fold in the presence of hGST A4-4, compared to the condition without enzyme. Thus, the 1400- and 7500-fold increases in the presence of hGSTs cannot be explained exclusively in terms of higher availability of GS^-^, suggesting the existence of additional mechanisms of catalysis. Generally, in enzymes, catalysis is supported by interactions established between active site amino acid residues and substrates. These interactions are responsible for stabilizing charges, for limiting the movement of the substrates and orienting them adequately, and for restricting the access of water to the active site. This resulting binding energy is maximized in the transition state and is used to lower the activation energy of the reaction, leading to products (62).

In this regard, the crystal structure of hGST M1-1 with the GS-10-NO_2_-OA adduct bound within the active site was solved, providing a snapshot of the residues involved in substrate binding and catalysis, and giving valuable structure-activity information. The structure reported herein is the first one of a GST with a nitro fatty acid derivative, thus the starting point for further studies on enzyme-substrate specificity, of particular relevance considering the variety of GST isoforms and NO_2_-FAs. Some residues likely relevant for catalysis were identified (Fig. 8). The hydrogen bond between Y7 and GSH has been previously observed and proposed to lower the p*K*_*a*_ of bound GSH, thus favoring the availability of the nucleophile (thiolate) (40, 43, 59, 61). Mutation of this residue to phenylalanine (Y7F) led to a dramatic decrease in *k*_cat_ measured with CDNB and GSH (59, 61). Regarding Y116, which interacts with the nitro group, previously reported crystallographic (60) and kinetic data (61) suggest that it participates in the binding of the electrophilic substrate and in some of the chemical steps of the reaction. It would be interesting to explore in future studies the importance of this residue in the interaction with NO_2_-OA.

Similar interactions to those carried out by these two tyrosines (Y7 and Y116) can be suggested in the model of hGST A4-4 bound to GS-10-NO_2_-OA (Fig. 9). In this case, it is Y9 that plays the role of catalytic residue, adequately positioned to interact with the glutathionyl sulfur. Regarding Y212 in hGST A4-4, it has been previously reported to participate in the binding of alkenals (44, 63), thus supporting our hypothesis that it fulfills an analogous role to Y116 in hGST M1-1.

One of the main differences between the enzyme–adduct complexes of hGST M1-1 and A4-4 appears to occur in the proximity of the fatty acid’s carboxy terminus (Fig. 10). In particular, the presence of R221 in hGST A4-4 provides a positive charge ideally positioned to establish a strong salt bridge with the carboxylate of the fatty acid. This interaction is likely favored by the length of the carbon chain, suggesting that the binding of shorter NO_2_-FAs would not be equally efficient. In this regard, the catalysis of hGST A4-4 with 4-hydroxy alkenals of different lengths presented increased *k*_cat_/*K*_m_ with higher number of carbons; for example, *k*_cat_/*K*_m_ was 83-fold higher for 4-hydroxydecenal than for 4-hydroxypentenal (45). Thus, the presence of R221 is likely key to explain why hGST A4-4 catalyzes 6-fold faster than hGST M1-1.

Finally, the crystal structure of hGST M1-1 also suggested that, as there is a fixed and conserved site for GSH binding and a very well-defined site to accommodate and interact with the nitro group, the enzyme can probably only bind and catalyze the reaction using the (*E*)-isomer of NO_2_-OA leading to the formation of the product with (*R*,*S*) configuration at the chiral carbons 20 and 31, that was observed herein. Notably, (*E*)-isomers of nitroalkenes were reported to be thermodynamically more stable than (*Z*)-isomers (12, 64).

Altogether, the data presented in this article show that at least two cytosolic hGSTs are able to catalyze the addition of GSH to NO_2_-OA, a prototypical NO_2_-FA used in most preclinical models (5), which is being clinically developed and evaluated on a phase II clinical trial on obese asthmatics. Given the high abundance of hGSTs and GSH in cells, it is likely that the catalyzed reaction can occur *in vivo*. The cytosolic concentration of GSH is usually in the high mM range, hence hGSTs are likely saturated with GSH. The concentrations of the second substrates of hGSTs are probably low and variable. In the case of NO_2_-OA, following oral administration, plasma concentrations of NO_2_-OA were reported to be in the low μM range (0.2–0.5 μM) for up to 16 h (64). This is a fraction of the pool of NO_2_-OA, as it is mostly esterified in triglycerides. As triglycerides found in chylomicrons are specifically hydrolyzed in tissues, high local concentrations (μM range) can be achieved in capillaries. Intravenous administration led to higher concentrations of NO_2_-OA in plasma (∼12 μM) that decayed to less than 1.5 μM after the first hour (65). Thus, the intracellular concentration of free NO_2_-OA would probably be in the nM range and likely below the corresponding *K*^NO^_2_^-OA^. Hence, an increase in the concentration of NO_2_-OA will probably lead to an increase in its rate of consumption by hGSTs. Among several possible second substrates, a particular GST will prefer to react with the substrate that presents the higher *k*_cat_/*K*_m_ (specificity constant) multiplied by the substrate concentration. In this regard, the *k*_cat_/*K*^NO^_2_^-OA^ for hGST M1-1 is 8.8 × 10^4^ M^-1^ s^-1^ at pH 7.4 (4-fold lower than with CDNB), while for hGST A4-4, the *k*_cat_/*K*^NO^_2_^-OA^ is 4.8 × 10^5^ M^-1^ s^-1^ (80-fold higher than with CDNB) (Table S1). Thus, depending on the presence of alternative second substrates, NO_2_-OA may be a competitor for these hGSTs, especially for hGST A4-4.

Considering that hGSTs represent ∼10% of cytosolic proteins in mammalian tissues (66) and that the concentration of total cytosolic proteins is ∼150 mg/ml (67), ∼15 mg/ml hGSTs (roughly 0.6 mM) are expected in the cytosol. Here, we determined acceleration values of (8.3 ± 0.9) × 10^5^ M^-1^ for hGST M1-1 and (4.7 ± 0.7) × 10^6^ M^-1^ for hGST A4-4 and NO_2_-OA at 2 mM GSH. These values suggest that the reaction with GSH can be accelerated ∼500 times by hGST M1-1 and ∼3000 times by hGST A4-4, in the cell.

It is important to bear in mind that the expression of different isoforms of these enzymes is tissue-specific (43). This becomes relevant in a physiological context, considering that the effects of NO_2_-OA will depend on hGSTs’ activities, concentration, and specificity. It should also be noted that the expression of hGSTs is regulated by Nrf2 which is activated by NO_2_-OA (68). These variables must be considered when designing experiments to probe their role in NO_2_-OA metabolism. Bates et al. (38) observed an attenuation on the activation of PPARγ induced by NO_2_-OA in breast cancer cells (MCF7) overexpressing hGST M1-1, hGST A1-1, or hGST P1-1. This result suggests that an increase in hGST levels could lead to enhanced rates of NO_2_-OA inactivation affecting NO_2_-OA’s signaling actions. Thus, this aspect should be taken into consideration in pharmacokinetic evaluations.

Overall, our results increase our understanding of the metabolism of NO_2_-FAs and expand the repertoire of known substrates for GSTs.

## Experimental procedures

### Reagents

Reagents were obtained from Merck or Applichem unless specified otherwise. All the solvents used were HPLC grade or higher. Recombinant hGST M1-1, M2-2, M4-4, A4-4, and P1-1 used only for HPLC-ESI-MS/MS screening experiments were acquired in Oxford Biomedical Research. For the main experiments, hGST M1-1 and hGST A4-4 were recombinantly expressed and purified in-house as described in “Expression and purification of hGSTs.” Stock solutions of CDNB were prepared in ethanol. GSH stocks were prepared in phosphate buffer (20 or 100 mM, pH 7.4) except for the p*K*_*a*_ determination experiments, where they were prepared in water. The equimolar mixture of 9- and 10-NO_2_-OA was synthesized using the nitroselenation reaction (13) and 10-NO_2_-OA was synthesized following a nitro-aldol condensation (12). NO_2_-OA stocks (either 10-NO_2_-OA or the equimolar mixture of 9- and 10-NO_2_-OA) are *E* isomers (64). Working solutions of NO_2_-OA were prepared in methanol or DMSO. The concentration of NO_2_-OA was determined from absorbance measurements at 259 nm using an absorbance coefficient of 4500 M^-1^ cm^-1^ in methanol (manuscript in preparation).

### Reaction mixtures for HPLC-ESI-MS/MS analysis of hGST activity

For screening purposes, five commercially available cytosolic hGSTs were tested for their ability to catalyze the formation of adducts: hGST M1-1, M2-2, M4-4, A4-4, and P1-1. Reactions were started by mixing GSH (200 μM) with the equimolar mixture of 9- and 10-NO_2_-OA (2 μM) or purified 10-NO_2_-OA (2 μM), in the absence or presence of enzyme (0.07 μM), in phosphate buffer (20 mM, pH 7.4, 25 °C). After 5 min, the reactions (50 μl) were stopped by the addition of 150 μl of acetonitrile containing 1% (v/v) acetic acid. To obtain time courses of the reaction, in-house recombinant hGST M1-1 and A4-4 were used. GSH (200 μM) was mixed with 10-NO_2_-OA (2 μM), in the absence or presence of hGST M1-1 or A4-4 (0.07 μM). Aliquots (50 μl) were taken at different time points (10, 15, 20, 25, 30, 35, 40, 45 s) and the reactions were stopped as described.

### HPLC-ESI-MS/MS analysis of GS-NO_2_-OA

Samples were resolved on a reversed-phase HPLC column (Luna C18(2), 5 μm particle size, 2 × 100 mm, Phenomenex), at 0.65 ml/min flow rate using water, 0.1% (v/v) formic acid (solvent A), and acetonitrile, 0.1% (v/v) formic acid (solvent B). Samples (10 μl) were loaded at 20% B for 0.5 min and eluted by increasing B to 85% over 13 min, followed by 2 min of 100% B. MS/MS characterization of the GS-NO_2_-OA adducts was performed using a triple quadrupole mass spectrometer AB5000 (Sciex) with electrospray ionization (ESI) source in the positive ion mode with the following settings: source temperature, 550 °C; curtain gas 40; ionization spray voltage 5500; GS1, 50; GS2, 55; declustering potential, 70 V; collision energy, 17 V; collision cell exit potential, 5 V. The following MRM transition was used for GS-NO_2_-OA: 635.3/506.2 (69). The total area under the peak was determined.

### Expression and purification of hGSTs

#### Human glutathione transferase M1-1 (hGST M1-1)

The ORF of hGST M1-1 (X08020.1) was cloned (GenScript) into a pET22b plasmid between NdeI and BamHI restriction sites, and competent BL21(DE3) cells were transformed. Luria-Bertani medium supplemented with ampicillin (200 μg/ml) was inoculated with an overnight preculture and cultivated at 37 °C. When the optical density at 600 nm (OD_600_) reached 0.6 to 0.8, expression was induced with IPTG (0.4 mM) and cultures were further incubated at 37 °C for 3 h. The culture was centrifuged, and the pellet was resuspended in lysis buffer (PBS pH 7.0, 50 μM phenylmethylsulfonyl fluoride, 10 μg/ml aprotinin, 5 μM pepstatin A, 10 μg/ml DNAse, 1 mg/ml lysozyme, and 1% Triton X100). After sonication, the soluble fraction was loaded into a GSH-Sepharose (GSTprep, Cytiva) affinity chromatography column equilibrated with PBS pH 7.0 and further washed with the same buffer. Elution was performed with 10 mM GSH in Tris buffer (50 mM, pH 8.0). The eluted fraction was then loaded on a SEC column (HiLoad 16/600 Superdex 200, Cytiva) equilibrated with Tris buffer (50 mM, pH 8.0, 150 mM NaCl) on an AKTA-Prime Plus FPLC system. Protein concentration was determined by absorbance at 280 nm using the extinction coefficient (ɛ = 40,130 M^-1^ cm^-1^) calculated with ProtParam (Expasy) from the hGST M1-1 primary sequence (25,711 Da) and expressed as monomer concentration. The thiol content of hGST M1-1 (15 μM) was measured using DTNB (460 μM) in phosphate buffer (100 mM, pH 7.4, 0.1 mM diethylenetriaminepentaacetic acid (DTPA)), following the absorbance at 412 nm for 40 min (25 °C) (51). The purity of the protein was assessed by SDS-PAGE under reducing conditions. The identity was confirmed by peptide mass fingerprinting of the tryptic digest using matrix assisted laser desorption ionization-time of flight mass spectrometry (Institut Pasteur, Montevideo). The specific activity was determined using GSH and CDNB as substrates (see “hGST activity measurements” section).

#### Human glutathione transferase A4-4 (hGST A4-4)

The ORF of hGST A4-4 (Y13047.1) was cloned (GenScript) into a pET-Trx1b vector (kindly provided by Dr Günther Stier, EMBL-Heidelberg) between NdeI and BamHI restriction sites, immediately downstream of the sequence encoding a His-tagged Trx1 (*E*.*coli*) and a cleavage site for the TEV protease. Briefly, competent BL21(DE3) cells were transformed. 2 YT medium (16 g/L tryptone, 10 g/L yeast extract, 5 g/L NaCl) supplemented with kanamycin (50 μg/ml) was inoculated with an overnight preculture and grown at 37 °C until reaching 0.6 to 0.8 OD_600_. Expression was induced with IPTG (1 mM), and cultures were further incubated overnight at 20 °C. Culture centrifugation, lysis, sonication, and GSH-Sepharose chromatography were performed as described for hGST M1-1. The eluted fraction of this chromatography, corresponding to the Trx1-hGST A4-4 fusion, was then incubated with DTT (1 mM) and a His-tagged TEV protease (1:20 TEV protease to Trx1-hGST A4-4 ratio) for 2 h at 4 °C and dialyzed overnight against Tris buffer (50 mM, pH 8.0, 500 mM NaCl). As both Trx1 and TEV protease have a HisTag, an immobilized metal ion affinity chromatography was performed to separate them from hGST A4-4. The column (HisTrap, Cytiva) was equilibrated with Tris buffer (50 mM, pH 8.0, 500 mM NaCl); hGST A4-4 was collected from the flow through and then loaded on a SEC column. SEC was performed as for hGST M1-1. Protein concentration was determined by the absorbance at 280 nm using the extinction coefficient (ɛ = 17,420 M^-1^ cm^-1^) determined with ProtParam (Expasy) using the hGST A4-4 primary sequence (25,761 Da) and expressed as monomer concentration. SDS-PAGE, peptide mass fingerprinting, and activity measurements were performed as for hGST M1-1.

### hGST activity measurements

GSH (4 mM) and CDNB (1 mM), were mixed with hGST M1-1 (3–4 nM) or hGST A4-4 (40–80 nM) in phosphate buffer (100 mM, pH 7.4, 0.1 mM DTPA). Product formation was followed at 340 nm (ɛ = 9.6 mM^-1^ cm^-1^) (70) for 1 min at room temperature. The uncatalyzed reaction rate was also monitored in mixtures without enzyme and subtracted from the enzyme-catalyzed reaction. Specific activity was expressed as μmoles of product formed min^-1^ (mg protein)^-1^.

### HPLC-UV-Vis assessment of the reaction between NO_2_-OA and GSH

GSH (2 mM) was mixed with 10-NO_2_-OA (68 μM) in phosphate buffer (20 mM, pH 7.4, 25 °C). Aliquots were taken at different time points (12 s, 32 s, and 3 min) and the reactions were stopped with the addition of 20% acetonitrile, 7% formic acid (v/v, final concentrations). Time zero corresponded to 10-NO_2_-OA before GSH addition. In the presence of enzyme (2.5 μM hGST M1-1 or 1 μM hGST A4-4), the reaction was analyzed at 12 s. Samples (100 μl) were resolved in an HPLC (Agilent Infinity 1260) using the same column and chromatographic method used for HPLC-ESI-MS/MS experiments. Absorbance was registered at 260 nm, and spectra were obtained using a diode array detector.

### Stopped-flow kinetic studies of the reaction between NO_2_-OA and GSH

10-NO_2_-OA or the equimolar mixture of 9- and 10-NO_2_-OA (20 μM) were reacted with GSH (2 mM) in the absence or presence of increasing concentrations of hGST M1-1 (0.5–2.5 μM) or A4-4 (0.15–1.0 μM), in phosphate buffer (100 mM, pH 7.4, 0.1 mM DTPA, 25 °C). NO_2_-OA consumption was followed at 285 nm using a stopped-flow spectrophotometer (Applied Photophysics SX20) (31) with monochromator slits at 0.2 mm. A control in the absence of GSH was included for both enzymes.

### p*K*_*a*_ determination of GSH bound to hGSTs

The initial rate of the reaction between GSH and CDNB in the absence and presence of enzyme was measured at different pHs (5–10.2), using a three-component buffer of constant ionic strength (0.1 M 2-(N-morpholino)-ethane sulfonic acid (MES), 0.052 M Tris, 0.052 M ethanolamine) (54). Briefly, GSH (2 mM), CDNB (75 μM), and hGST M1-1 (1.4 nM) or A4-4 (14 nM) were mixed, and the increase in absorbance at 340 nm corresponding to product formation was followed (25 °C). The pH of the reaction mixtures was measured at the end of the experiment. The initial rate of the uncatalyzed reaction (in the absence of enzyme) was subtracted from the initial rate of the catalyzed reaction at each pH (52).

### Crystallographic determination of the structure of hGST M1-1 in complex with GS-10-NO_2_-OA

The GS-10-NO_2_-OA adduct was synthesized by mixing GSH (5 mM) with 10-NO_2_-OA (200 μM) in Tris buffer (50 mM, pH 8.0, room temperature). Equal volumes of the adduct and hGST M1-1 (5 mg/ml in 50 mM Tris buffer, pH 8.0, 150 mM NaCl) were mixed, incubated for 30 min in ice, concentrated to achieve a final protein concentration of 15 mg/ml, and flash frozen in liquid N_2_. Crystallization experiments and *in situ* room temperature data collection were performed at beamline VMXi (Diamond Light Source). Crystals were obtained at 20 °C in 96-well In Situ-1 plates (MiTeGen) by mixing 100 nl of protein with 50 nl of reservoir (0.1 M Hepes pH 7.5, 25% (w/v) PEG 3350). X-ray diffraction data from eight crystals were collected and processed with xia2.multiplex (71). The crystal structure was determined using initial phases from an isomorphous structure (PDB 1XW6 (61)), from which ligands and water molecules were previously eliminated. Buster/TNT (72) was employed to refine the atomic model, iterating with manual model building with Coot (73). Validation was done with MolProbity (74) previous to PDB deposition.

### Modeling of hGST A4-4 structure in complex with GS-10-NO_2_-OA

A model of hGST A4-4 in complex with the GS-10-NO_2_-OA adduct was prepared by structural superposition of the hGST A4-4 crystal structure (PDB 3IK7, (47)) and the coordinates of the hGST M1-1 in complex with GS-10-NO_2_-OA obtained in this work. The structural alignment was performed for each monomer separately. Classical parameters corresponding to the GS-10-NO_2_-OA adduct were derived using standard protocols (75). The hGST A4-4 dimeric complex was then subjected to a two-step energy minimization process by first minimizing only the ligand and then the whole system. The Sander module of the Amber package (76) was used for computing the minimization calculations, while the ff14SB force field (77) was considered for every protein residue. Structural analysis and molecular drawings were performed with VMD (78) and PyMol (https://www.pymol.org/support.html).

## Data availability

All data are contained within the manuscript and supporting information. hGST M1-1 crystal structure was deposited in the PDB (8VOU).

## Supporting information

This article contains supporting information (56, 57).

## Conflict of interest

F. J. S. has financial interest in Creegh Pharmaceuticals Inc and Furanica Inc. All other authors declare that they have no conflicts of interests with the contents of this article.

## Supporting information

Supplemenatry Materials
