# Supplementary material for: Human glutathione transferases catalyze the reaction between glutathione and nitrooleic acid
Source: J Biol Chem. 2025 Feb 28;301(4):108362. doi: 10.1016/j.jbc.2025.108362 (PMC11999266; doi:10.1016/j.jbc.2025.108362)
Supplement: Supplemenatry Materials [file mmc1.docx]

Supplementary information

Human glutathione transferases catalyze the reaction between glutathione and nitrooleic acid

Martina Steglich^1,2,3^, Nicole Larrieux^4^, Ari Zeida^2,5^, Joaquín Dalla Rizza^4^, Sonia Salvatore^6^, Mariana Bonilla^7^, Matías N. Möller^2,8^, Alejandro Buschiazzo^4^, Beatriz Alvarez^1,2^, Francisco J. Schopfer^6,9,10^, Lucía Turell^1,2,^*

^1^Laboratorio de Enzimología, Instituto de Química Biológica, Facultad de Ciencias, Universidad de la República, Montevideo, Uruguay.

^2^Centro de Investigaciones Biomédicas (CEINBIO), Universidad de la República, Montevideo, Uruguay

^3^Graduate Program in Chemistry, Facultad de Química, Universidad de la República, Montevideo, Uruguay

^4^Unidad de Cristalografía de Proteínas, Institut Pasteur de Montevideo, Montevideo, Uruguay.

^5^Departamento de Bioquímica, Facultad de Medicina, Universidad de la República, Montevideo, Uruguay.

^6^Department of Pharmacology and Chemical Biology, University of Pittsburgh School of Medicine, Pittsburgh, United States.

^7^Laboratorio de Biología Redox de Tripanosomas, Institut Pasteur de Montevideo, Montevideo, Uruguay.

^8^Laboratorio de Fisicoquímica Biológica, Instituto de Química Biológica, Facultad de Ciencias, Universidad de la República, Montevideo, Uruguay.

^9^Pittsburgh Heart, Lung and Blood Vascular Medicine Institute, University of Pittsburgh, Pittsburgh, PA, USA.

^10^Pittsburgh Liver Research Center, University of Pittsburgh, Pittsburgh, PA, USA.

*Correspondence to: Lucía Turell, Laboratorio de Enzimología, Instituto de Química Biológica, Facultad de Ciencias, Universidad de la República, Iguá 4225, Montevideo, 11400, Uruguay.

E-mail address: lturell@fcien.edu.uy (L. Turell)

**Materials included:** Figure S1, Figure S2, Figure S3, Figure S4, Figure S5, Figure S6, Table S1, Figure S7, Figure S8, Figure S9, Figure S10 and Figure S11.

**
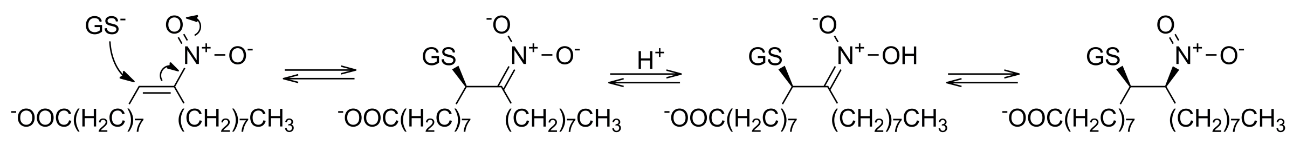
**

**Figure S1. Mechanism of the reaction between (*E*)-10-NO_2_-OA and GSH.** In the final product, the chiral carbons bound to the glutathionyl and nitro groups are drawn with *R* and *S* configurations, respectively, according to the crystal structure of hGST M1-1 in complex with the GS-NO_2_-OA adduct (this work).


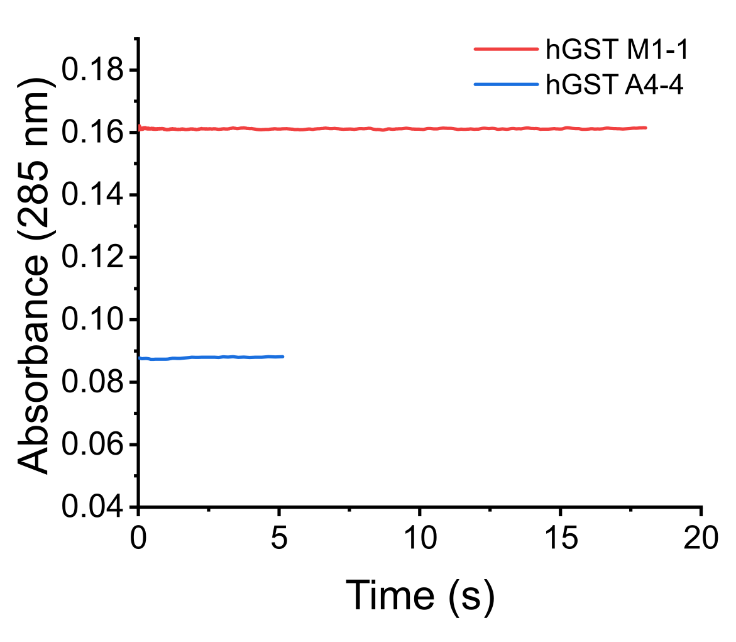


**Figure S2. Control of NO_2_-OA and hGSTs in the absence of GSH.** An equimolar mixture of 9- and 10-NO_2_-OA (20 µM) was mixed with hGST M1-1 (2.5 µM) or hGST A4-4 (1 µM) in phosphate buffer (100 mM pH 7.4, 0.1 mM DTPA, 25 °C). Absorbance at 285 nm was registered using a stopped-flow spectrophotometer. The concentration of enzyme used corresponds to the highest hGST concentration used in the presence of GSH (Figs. 6A and 6B).


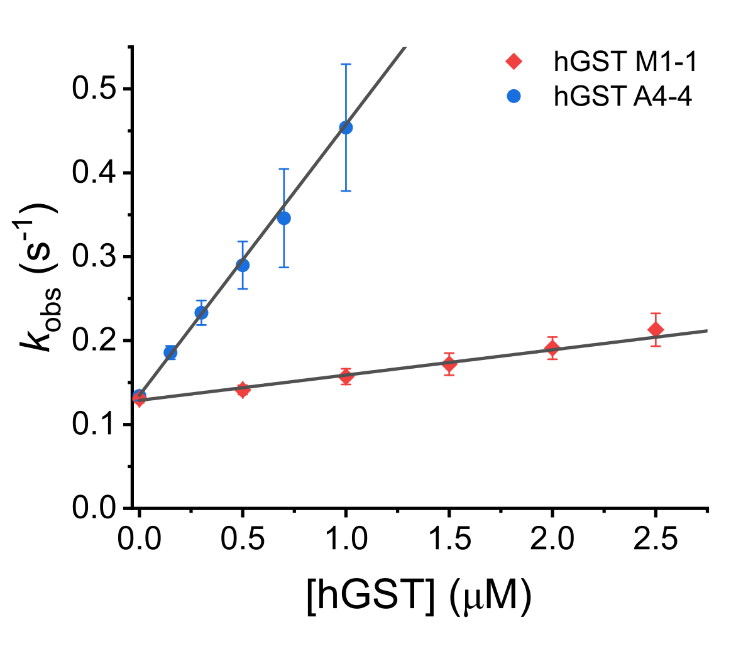


**Figure S3. Kinetics of the reaction between 10-NO_2_-OA and GSH catalyzed by hGST M1-1 and hGST A4-4.** 10-NO_2_-OA (20 µM) was mixed with GSH (2 mM) in the absence (uncatalyzed) or presence of hGST M1-1 (0.5-2.5 µM) and hGST A4-4 (0.15-1.0 µM), in phosphate buffer (100 mM pH 7.4, 0.1 mM DTPA, 25 °C). The *k*_obs_ values were determined from the fits of the non-averaged data as in Fig. 6 and plotted against hGST concentration. The symbols represent the mean ± standard deviation of n = 12 (3 independent experiments with 4 repetitions each). Some error bars are smaller than the symbols.

**
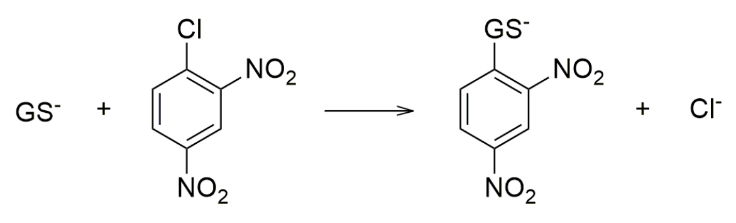
**

**Figure S4. Reaction between the thiolate of glutathione (GS^-^) and CDNB.**


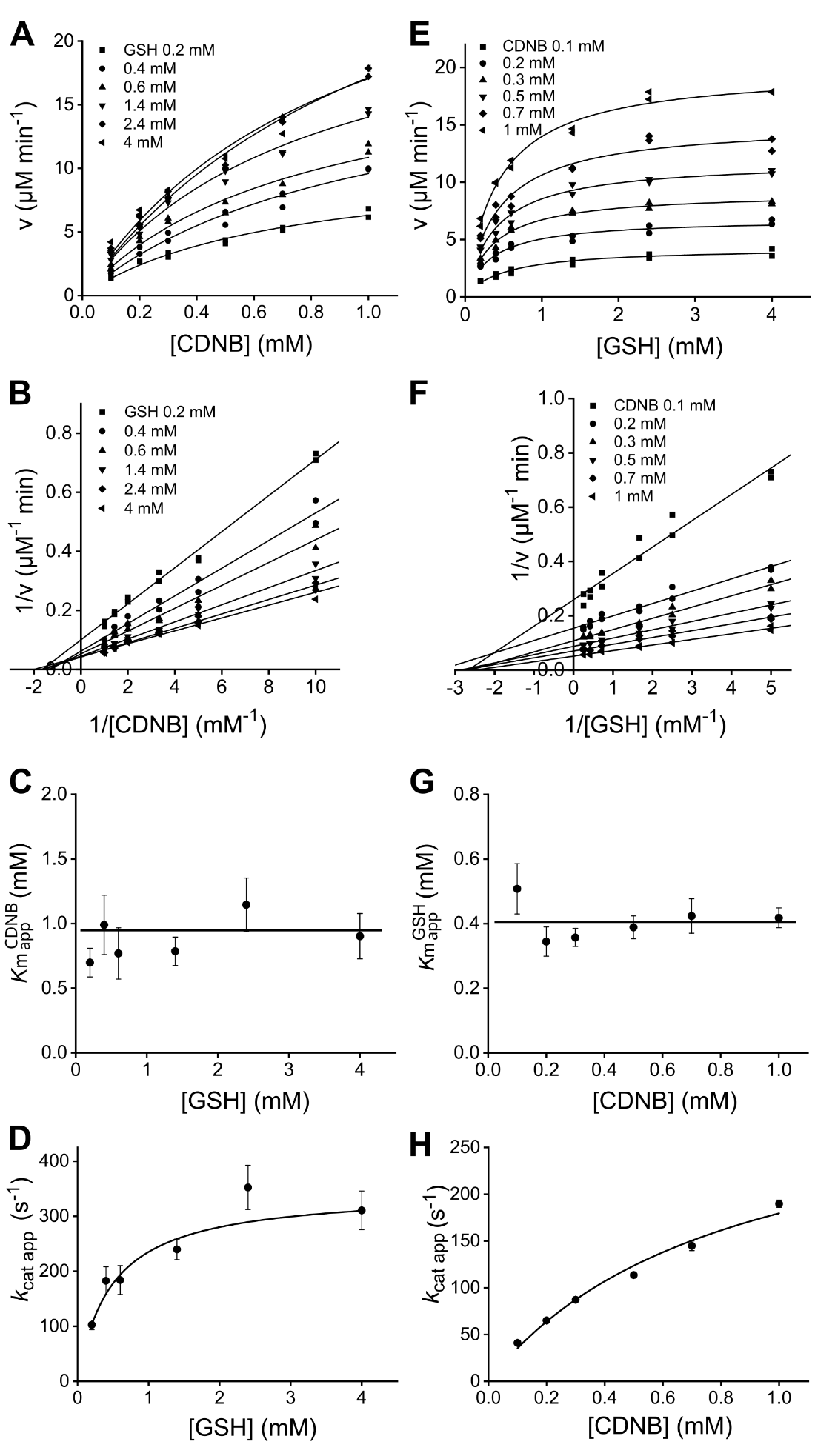


**Figure S5.** **Two-substrate mechanism of hGST M1-1 with CDNB and GSH.** The initial rates of the reaction between CDNB and GSH in the presence of hGST M1-1 (1.7 nM) were determined by following the absorbance at 340 nm under conditions where one substrate was held constant while the other one was varied (CDNB 0.1–1 mM, GSH 0.2–4 mM, 100 mM phosphate buffer, pH 7.4, 0.1 mM DTPA, 25 °C). (A, E) Initial rate versus concentration plots were fitted to Michaelis-Menten-like hyperbolic equations to obtain apparent kinetic parameters (*K*m_app_ and *k*_cat_). (B, F) The data were also plotted using the Lineweaver-Burk linearization. (C, G) *K*m_app_ and (D,H) *k*_cat_ values were plotted against the concentration of the fixed substrate.


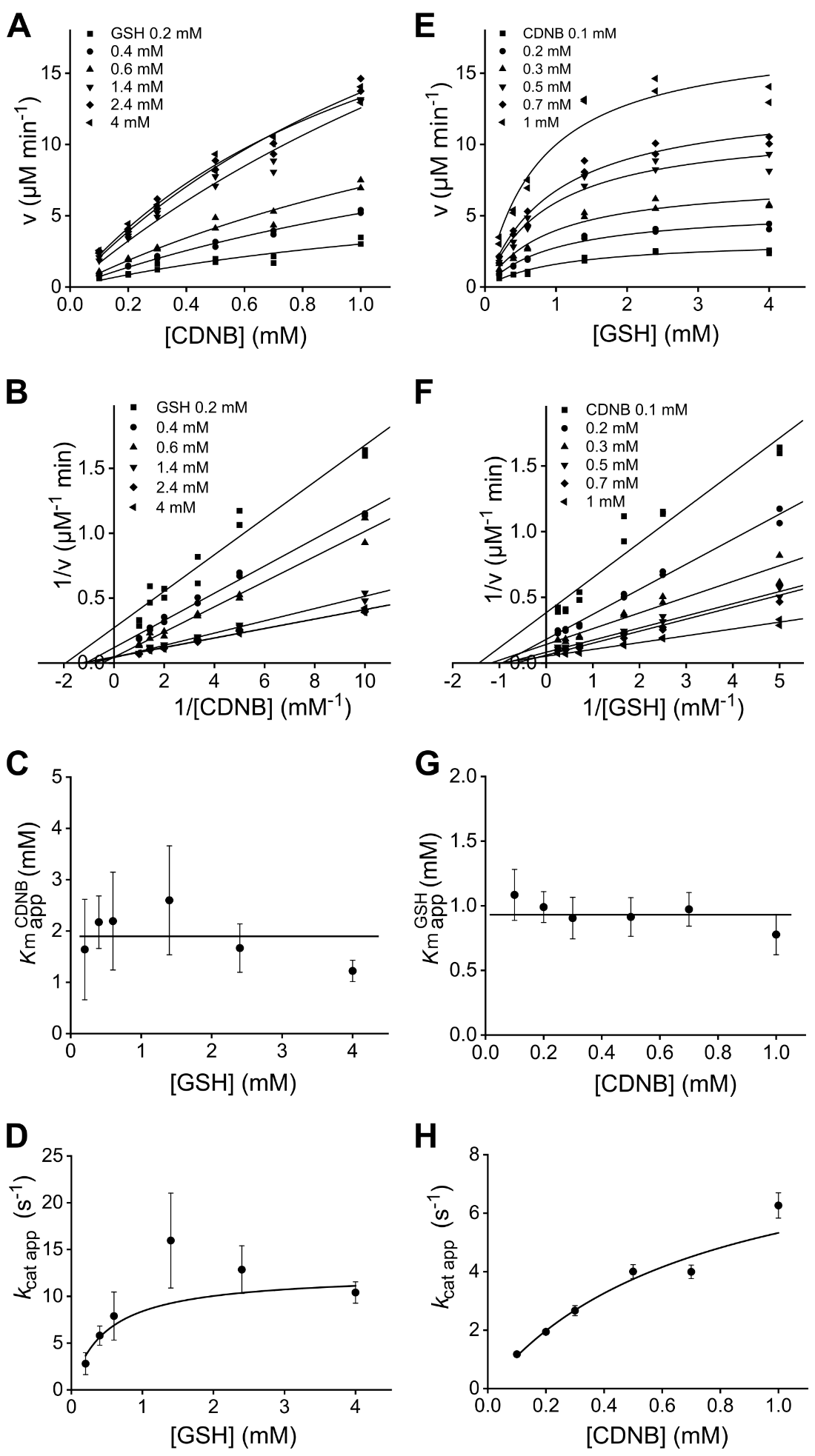


**Figure S6.** **Two-substrate mechanism of hGST A4-4 with CDNB and GSH.** The initial rates of the reaction between CDNB and GSH in the presence of hGST A4-4 (47 nM) were determined by following the absorbance at 340 nm under conditions where one substrate was held constant while the other one was varied (CDNB 0.1–1 mM, GSH 0.2–4 mM, 100 mM phosphate buffer, pH 7.4, 0.1 mM DTPA, 25 °C). (A, E) Initial rate versus concentration plots were fitted to Michaelis-Menten-like hyperbolic equations to obtain apparent kinetic parameters (*K*m_app_ and *k*_cat_). (B, F) The data were also plotted using the Lineweaver-Burk linearization. (C, G) *K*m_app_ and (D, H) *k*_cat_ values were plotted against the concentration of the fixed substrate.

**Table S1.** Kinetic parameters (25°C, pH 7.4) for hGST M1-1 and hGST A4-4 with GSH and CDNB as substrates obtained from the data shown in Figures S5 and S6 assuming a rapid-equilibrium random mechanism ^a^.

|  | hGST M1-1 | hGST A4-4 |
| --- | --- | --- |
| *K*^CDNB^ (mM) | 0.9 ± 0.2 | 1.9 ± 0.5 |
| *K*^GSH^ (mM) | 0.41 ± 0.06 | 0.9 ± 0.1 |
| *k_cat_* (s^-1^) | 337 ± 13 | 11 ± 2 |
| *k_cat_*/*K*^CDNB^ (M^-1^ s^-1^) | 3.7 × 10^5^ | 5.8 × 10^3^ |
| *k_cat_*/*K*^GSH^ (M^-1^ s^-1^) | 8.2 × 10^5^ | 1.2 × 10^4^ |

^a^ The hyperbolic behavior of the initial rate *versus* substrate concentration plots obtained with CDNB and GSH (Figs. S5 and S6) is consistent with a rapid equilibrium random model, since the steady-state random model yields non-hyperbolic equations (56, 57). However, deviations from hyperbolic behavior may be difficult to detect. Considering that the dissociation constants obtained for GSH in our experiments are relatively high and that the crystal structure suggests that GSH interacts with the enzyme through several simultaneous interactions, compatible with a lower dissociation constant, it is possible that the enzymes follow a random sequential steady-state mechanism.


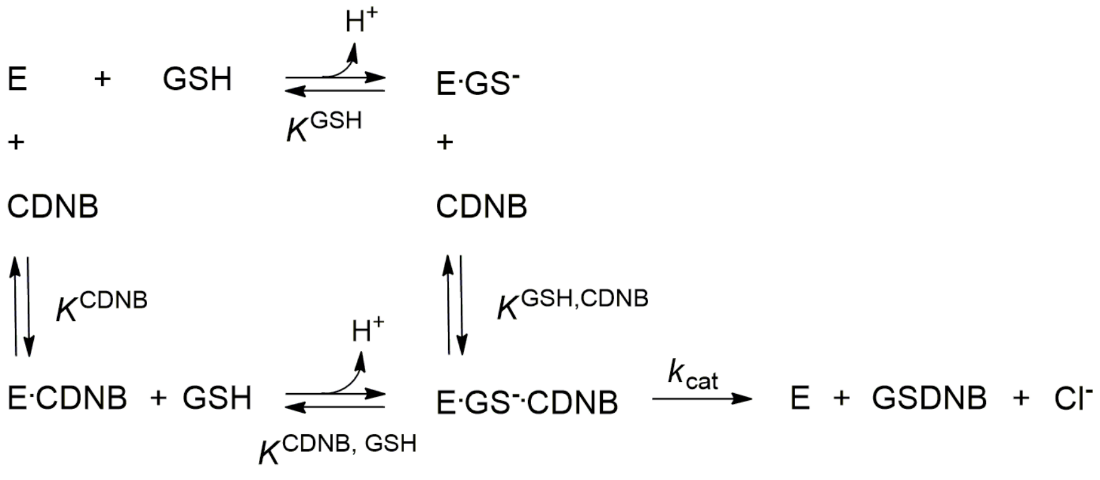


**Figure S7.** **Random sequential mechanism for the canonical reaction of hGST M1-1 and hGST A4-4, represented as E, with CDNB and GSH as substrates.**

The rate of the reaction for a bisubstratic enzyme is described, in the case of the canonical reaction catalyzed by GST, by the general equation,

$v_{cat}=\frac{k_{cat}\left[ GST \right]_{T}\left[ GSH \right]\left[ CDNB \right]}{K^{GSH}K^{GSH,CDNB}+K^{GSH,CDNB}\left[ GSH \right]+K^{CDNB,GSH}\left[ CDNB \right]+\left[ GSH \right]\left[ CDNB \right]}$ (Eq. S1)

The binding of one substrate does not affect the binding of the other substrate (Figs. S5 and S6),

$K^{GSH}=K^{CDNB,GSH}$ (Eq. S2)

$K^{CDNB}=K^{GSH,CDNB}$ (Eq. S3)

Thus, the general equation can be rewritten,

$v_{cat}=\frac{k_{cat}\left[ GST \right]_{T}\left[ GSH \right]\left[ CDNB \right]}{K^{GSH}K^{CDNB}+K^{CDNB}\left[ GSH \right]+K^{GSH}\left[ CDNB \right]+\left[ GSH \right]\left[ CDNB \right]}$ (Eq. S4)

This can be extrapolated to NO_2_-OA,

$v_{cat}=\frac{k_{cat}\left[ GST \right]_{T}\left[ GSH \right]\left[ NO_{2}˗OA \right]}{K^{GSH}K^{NO_{2}˗OA}+K^{NO_{2}˗OA}\left[ GSH \right]+K^{GSH}\left[ NO_{2}˗OA \right]+\left[ GSH \right]\left[ NO_{2}˗OA \right]}$ (Eq. S5)

Assuming,

$\left[ GSH \right]>K^{GSH}$ (Eq. S6)

$\left[ NO_{2}˗OA \right]<K^{NO_{2}-OA}$ (Eq. S7)

Considering that NO_2_-OA and GSH also react in the absence of enzyme,

$v=v_{uncat}+v_{cat}$ (Eq. S8)

Finally, assuming that the reverse reaction is negligible,

$v=k_{on uncat}\left[ GSH \right]\left[ NO_{2}˗OA \right]+\frac{k_{cat}}{K^{NO_{2}-OA}} \left[ GST \right]\left[ NO_{2}˗OA \right]$ (Eq. S9)

**Figure S8. Derivation of equations 5 and 6.**


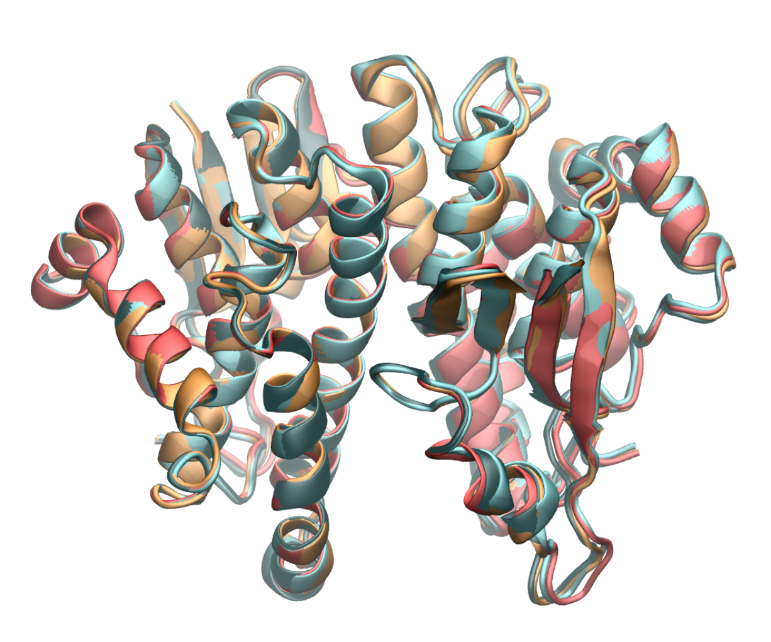


**Figure S9. Structural alignment of hGST M1-1 crystal structures.** The structure obtained in the present work (PDB 8VOU) represented in *grey* was aligned with previously reported structures of hGST M1-1 represented in *red* (PDB 7BEU), *cyan* (PDB 1XW6) and *orange* (PDB 1XWK) using VMD software.

**
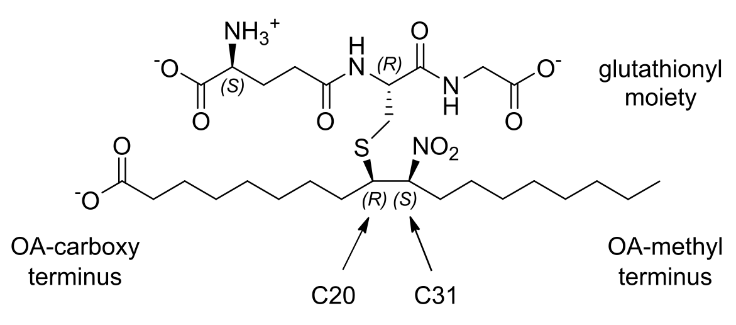
**

**Fig S10. Schematic representation of the GS-10-NO_2_-OA adduct.** The oleic acid (OA) carboxy and methyl termini, and the glutathionyl moiety of the adduct are highlighted. The chiral carbon atoms identified as C20 and C31 in the crystal structure are represented with their corresponding stereoisomerism (PDB 8VOU, this work), as well as the chiral carbons present in the glutathionyl moiety.

**
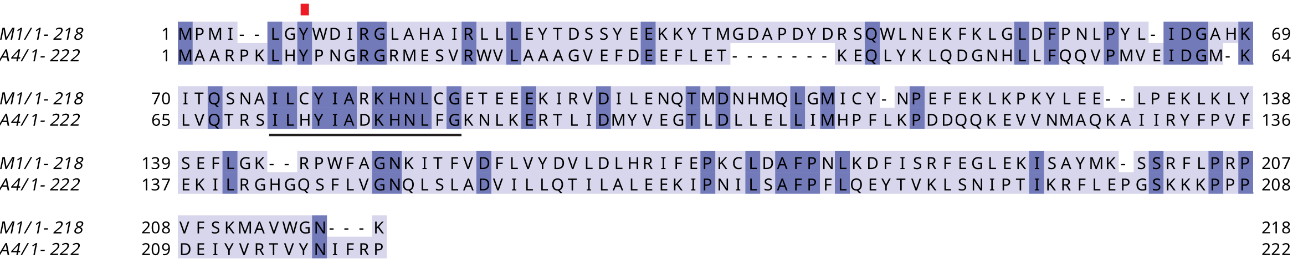
**

**Figure S11. Pairwise sequence alignment between hGST M1-1 and A4-4.** The alignment was performed using EMBOSS Stretcher. The catalytic tyrosine is marked with a red tag. Color shades highlight pairs of identical residues. The longest similar stretch (underlined) is located on helix α3 and corresponds to 76-**IL**C**YIA**R**KHNL**C**G**-88 according to hGST M1-1 numbering, with conserved residues highlighted in bold. These residues are engaged in packing α3 against helices α1 and α6, and the β sheet, of the same monomer, as well as with helix α4’ from the other monomer. Also, as a result of the alignment, 21.3% identity and 42.6% similarity scores were obtained among the two hGSTs.
